# *Candida albicans* mannans mediate *Streptococcus mutans* exoenzyme GtfB binding to modulate cross-kingdom biofilm development *in vivo*

**DOI:** 10.1371/journal.ppat.1006407

**Published:** 2017-06-15

**Authors:** Geelsu Hwang, Yuan Liu, Dongyeop Kim, Yong Li, Damian J. Krysan, Hyun Koo

**Affiliations:** 1 Biofilm Research Labs, Levy Center for Oral Health, Department of Orthodontics, School of Dental Medicine, University of Pennsylvania, Philadelphia, PA, United States of America; 2 Department of Pediatrics, Infectious Diseases and Microbiology & Immunology, University of Rochester Medical Center, Rochester, NY, United States of America; University of Birmingham, UNITED KINGDOM

## Abstract

*Candida albicans* is frequently detected with heavy infection by *Streptococcus mutans* in plaque-biofilms from children with early-childhood caries (ECC). This cross-kingdom biofilm contains an extensive matrix of extracellular α-glucans that is produced by an exoenzyme (GtfB) secreted by *S*. *mutans*. Here, we report that mannans located on the outer surface of *C*. *albicans* cell-wall mediates GtfB binding, enhancing glucan-matrix production and modulating bacterial-fungal association within biofilms formed *in vivo*. Using single-molecule atomic force microscopy, we determined that GtfB binds with remarkable affinity to mannans and to the *C*. *albicans* surface, forming a highly stable and strong bond (1–2 nN). However, GtfB binding properties to *C*. *albicans* was compromised in strains defective in *O*-mannan (*pmt4ΔΔ*) or *N*-mannan outer chain (*och1ΔΔ*). In particular, the binding strength of GtfB on *och1ΔΔ* strain was severely disrupted (>3-fold reduction vs. parental strain). In turn, the GtfB amount on the fungal surface was significantly reduced, and the ability of *C*. *albicans* mutant strains to develop mixed-species biofilms with *S*. *mutans* was impaired. This phenotype was independent of hyphae or established fungal-biofilm regulators (*EFG1*, *BCR1*). Notably, the mechanical stability of the defective biofilms was weakened, resulting in near complete biomass removal by shear forces. In addition, these *in vitro* findings were confirmed *in vivo* using a rodent biofilm model. Specifically, we observed that *C*. *albicans och1ΔΔ* was unable to form cross-kingdom biofilms on the tooth surface of rats co-infected with *S*. *mutans*. Likewise, co-infection with *S*. *mutans* defective in GtfB was also incapable of forming mixed-species biofilms. Taken together, the data support a mechanism whereby *S*. *mutans*-secreted GtfB binds to the mannan layer of *C*. *albicans* to promote extracellular matrix formation and their co-existence within biofilms. Enhanced understanding of GtfB-*Candida* interactions may provide new perspectives for devising effective therapies to disrupt this cross-kingdom relationship associated with an important childhood oral disease.

## Author summary

Early childhood caries is a severe form of tooth decay that affects toddlers and is clinically distinct from other forms of the disease. It is characterized by the frequent isolation of the commensal yeast *Candida albicans* and the bacterial pathogen *Streptococcus mutans* within caries-associated biofilms. This mixed-kingdom biofilm contains an extensive matrix of glue-like polymer of glucan that is generated by an enzyme (GtfB) secreted by *S*. *mutans*. The production of the glucan component of the biofilm matrix is promoted by the presence of *C*. *albicans*, creating a sticky accumulation of pathogenic biofilms. Here, we report that GtfB binds to mannoproteins on the outer surface of the *C*. *albicans* cell wall with remarkable strength. *C*. *albicans* strains with defects in mannoprotein biosynthesis have reduced levels of extracellular glucan polymer in the presence of *S*. *mutans*, and are unable to co-exist within biofilms. Accordingly, *C*. *albicans* strains lacking mannoproteins do not support cross-kingdom biofilm formation in an animal model of the disease. Detailed molecular studies of cross-kingdom biofilms can provide important information that may be useful in the development of new approaches for the prevention and treatment of this prevalent and costly oral disease of childhood.

## Introduction

*Candida albicans* is an opportunistic fungal pathogen found in various sites of the human body causing localized and systemic infections [1–3]. The ability of this organism to infect and cause diseases is associated with biofilm formation [4–6], often involving interactions with bacteria that can enhance the biofilm accumulation and virulence [7–10]. In the mouth, *C*. *albicans* is known to co-adhere with commensal streptococci (viridans group streptococci) that promote enhanced fungal carriage, leading to oral mucosal infection [11, 12]. However, several clinical studies also reveal that *C*. *albicans* is frequently detected with high numbers of *S*. *mutans* (a cariogenic pathogen) in plaque-biofilms formed on tooth surfaces of toddlers with early childhood caries (ECC) [13–15], an aggressive form of tooth decay characterized by protracted sugar consumption and rampant carious lesions [16, 17].

The clinical findings are intriguing because *C*. *albicans* does not co-adhere well with *S*. *mutans* in the absence of sucrose [18, 19], nor does it colonize teeth effectively on its own or cause severe caries in rodent models [20, 21]. Furthermore, *C*. *albicans* is usually absent in plaque-biofilms of healthy, ECC-free children [13–15]. However, previous *in vitro* studies revealed that secreted bacterial exoenzymes termed glucosyltransferases (Gtfs) can promote the co-existence between *S*. *mutans* and *C*. *albicans* within biofilms when clinical conditions are conducive of ECC (e.g. sugar-rich exposure). Gtfs produced by *S*. *mutans* can utilize dietary sucrose to produce exopolysaccharides (EPS) known as α-glucans, which forms the core of the extracellular matrix in cariogenic biofilms [22]. We found that *S*. *mutans*-derived GtfB is capable of binding to *C*. *albicans* cell surface and produce large amounts of extracellular α-glucans on the fungal surface [23]. These EPS formed *in situ* provide enhanced bacterial binding sites for *S*. *mutans* [18] that promote co-adhesion and mixed-species biofilm formation with glucan-rich matrix on apatitic surfaces [21, 24, 25]. Using a rodent model of dental caries, we observed that this interaction synergistically enhances the carriage of *S*. *mutans* and *C*. *albicans*, and the virulence of plaque-biofilms, leading to rampant caries *in vivo* [21]. The available data show a distinctive cross-kingdom association, which relies on a biochemical interaction mediated by a bacterial exoenzyme (and its product) attached to the fungal surface. This mechanism differs considerably from other bacterial-fungal co-adhesion found between *S*. *gordonii* (or *Staphylococcus aureus*) and *C*. *albicans*, which is based on cell-to-cell binding via adhesion-receptor interactions. To further understand the mechanistic basis of this biochemical interaction between GtfB and *C*. *albicans*, we were interested in identifying the fungal surface molecules to which GtfB binds, and thereby, mediate the development of bacterial-fungal biofilms on teeth.

The *C*. *albicans* cell wall is a dynamic and complex structure critical for maintaining the cell shape and immunogenicity [26]. As the major point of contact between the fungus and adhesins on microbial surfaces [27], several cell wall components have been shown to be important for adhesion with bacteria [28, 29] and biofilm formation [30–32]. Conversely, *C*. *albicans* cell wall components could also mediate binding of secreted metabolic byproducts and/or extracellular signaling molecules [33, 34]. Among three major components of the fungal cell wall (mannans, glucans and chitin), mannans are located at the most outer cell wall layer of *C*. *albicans* [32]. Thus, it is conceivable that these biomolecules are involved in the binding of GtfB exoenzyme to the fungal cell surface.

Here, we test the hypothesis that GtfB binds to mannoproteins in the cell-wall of *C*. *albicans*. We also explore the role of mannosylation in the development of mixed-species biofilms on apatitic surface. The data reveal that GtfB binds strongly to purified mannans, while *C*. *albicans* strains lacking genes critical for the biosynthesis of either *N*- or *O*-linked mannans showed severely reduced GtfB binding relative to wild type strains. Furthermore, mannoprotein-defective mutants were impaired in their ability to form mixed-species biofilms with *S*. *mutans* on apatitic surface as evidenced by a reduction in both α-glucan content and microbial population. This phenotype was independent of hyphae or other known fungal-biofilm regulators as *C*. *albicans efg1ΔΔ* (hyphae defective) or *bcr1ΔΔ* (key biofilm adhesion regulator) similarly bound GtfB and formed robust mixed-species biofilms. In addition, the mechanical stability of the defective biofilms was significantly weakened, resulting in near complete biomass removal by shear forces. Finally, these *in vitro* observations were confirmed *in vivo* using a rat biofilm model. Specifically, we observed that *C*. *albicans och1ΔΔ* was unable to form cross-kingdom biofilms on the tooth surface of rats co-infected with *S*. *mutans*. Likewise, co-infection with *S*. *mutans* defective in GtfB does not yield mixed-species biofilms *in vivo*. Altogether, our data strongly support a model whereby *S*. *mutans*-secreted GtfB binds to the mannan layer of *C*. *albicans* to promote the development of mixed-species biofilms. As such, our study provides mechanistic insights for the molecular basis of a cross-kingdom biofilm interaction associated with a prevalent and costly childhood disease.

## Results

### GtfB binding force measurements on *Candida albicans* mannosylation mutant strains

**Fig 1:**
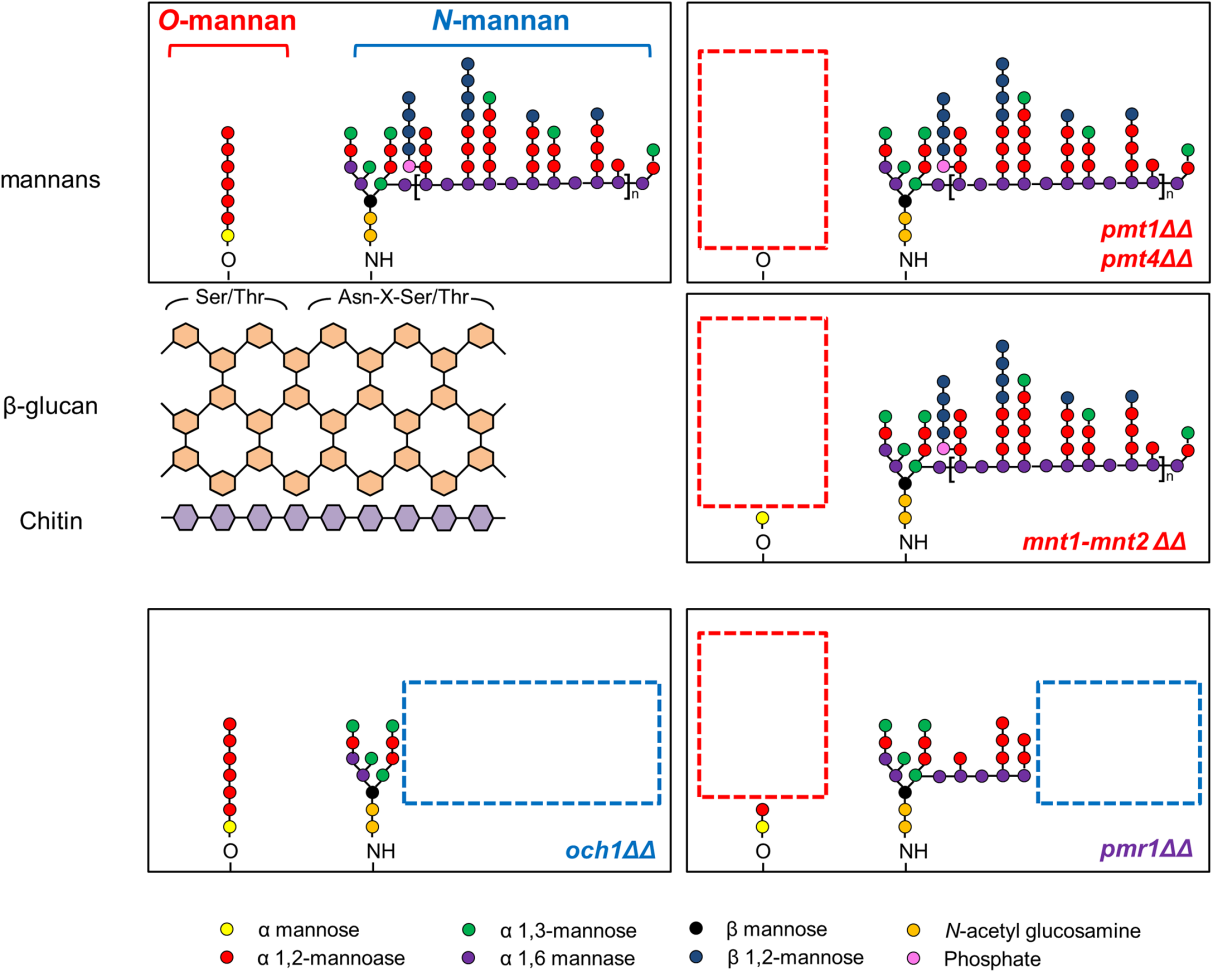
Cell wall mannoproteins structure of *Candida albicans* strains. Boxes with red and blue dotted lines describe the truncation of *O*- and *N*-linked mannans, respectively. Adapted from “Mannosylation in *Candida albicans*: role in cell wall function and immune recognition,” by Rebecca A. Hall and Neil A. R. Gow, 2013, Molecular Microbiology, 90(6), p. 1148 [26]. Copyright 2013 by the John Wiley & Sons Ltd. Adapted with permission.

Mannans are the major components of the outer most layer of *C*. *albicans* cell wall, and our preliminary studies indicate that GtfB can bind to purified mannans [21]. Thus, we investigated the potential role of mannosylated proteins in mediating GtfB binding and activity onto the fungal surface. To determine which of these polysaccharides may be critical for GtfB adhesive interactions, we selected well-characterized *C*. *albicans* mutants with specific truncations in the wild-type structures of *O-* and *N-*linked mannans (as summarized in Fig 1 and Table 1). We initially investigated the physical binding interactions between the exoenzyme and *C*. *albicans* cell surface via single-molecule atomic force microscopy (AFM). AFM tips functionalized with GtfB were used to measure the magnitude of force and force distribution/localization of GtfB binding to the surface of a single *C*. *albicans* cell as illustrated in S1 Fig.

**Table 1:** Summary of *C*. *albicans* strains used in this study.

Strains	Description	Cell wall	Reference
***C*. *albicans***			
SC5314	Prototrophic parent of CAI4	Normal	[35]
CAI4	*ura3Δ*::*imm434/ura3Δ*::*imm434*	Normal	[35]
NGY152	As CAI4 but *RPS1/rps1Δ*::*CIp10*	Normal	[36]
SN152	As CAI4 butarg4Δ/*arg4Δ leu2Δ/leu2Δ his1Δ/his1Δ IRO1/iro1Δ*::*imm434*	Normal	[37]
SPCa2 (*pmt1ΔΔ*)	*pmt1Δ*::*hisG/pmt1Δ*::*hisG*	Reduced *O*-mannan	[38]
SPCa6 (*pmt4ΔΔ*)	*pmt4Δ*::*hisG/pmt4Δ*::*hisG*	Reduced *O*-mannan	[38]
NGY355 (*pmr1ΔΔ*)	As CAI4 but *pmr1Δ*::*hisG/pmr1Δ*::*hisG*, *RPS1/rps1Δ*::*CIp10*	Reduced phosphomannan and *O*-mannan	[39]
NGY356(revertant of *pmr1ΔΔ*)	As CAI4 but *pmr1Δ*::*hisG/pmr1Δ*::*hisG*, *RPS1/rps1Δ*::*CIp10-PMR1*	Normal	[39]
NGY357 (*och1ΔΔ*)	As CAI4 but *och1Δ*::*hisG/och1Δ*::*hisG*, *RPS1/rps1Δ*::*CIp10*	Reduced phosphomannan and *N*-mannan	[40]
NGY358(revertant of *och1ΔΔ*)	As CAI4 but *och1Δ*::*hisG/och1Δ*::*hisG*, *RPS1/rps1Δ*::*CIp10-OCH1*	Normal	[40]
NGY337 (*mnt1-mnt2ΔΔ*)	As CAI4 but *mnt1-mnt2Δ*::*hisG/mnt1-mnt2Δ*::*hisG*	Reduced *O*-mannan	[41]
NGY335(revertant of mnt1-*mnt2ΔΔ*)	As CAI4 but *mnt1-mnt2Δ*::*hisG/mnt1-mnt2Δ*::*hisG*, *RPS10/rps10Δ*::*CIp10-MNT1*	Normal	[41]
NGY336(revertant of mnt1-*mnt2ΔΔ*)	As CAI4 but *mnt1-mnt2Δ*::*hisG/mnt1-mnt2Δ*::*hisG*, *RPS10/rps10Δ*::*CIp10-MNT2*	Normal	[41]
CAN33 (*efg1ΔΔ)*	As SN152 but *efg1Δ*::*HIS1 efg1Δ*::*LEU2*	Normal	[42]
CJN702 (*bcr1ΔΔ*)	As SN152 but *bcr1Δ*::*HIS1 bcr1Δ*::*LEU2*	Normal	[43]

**Fig 2:**
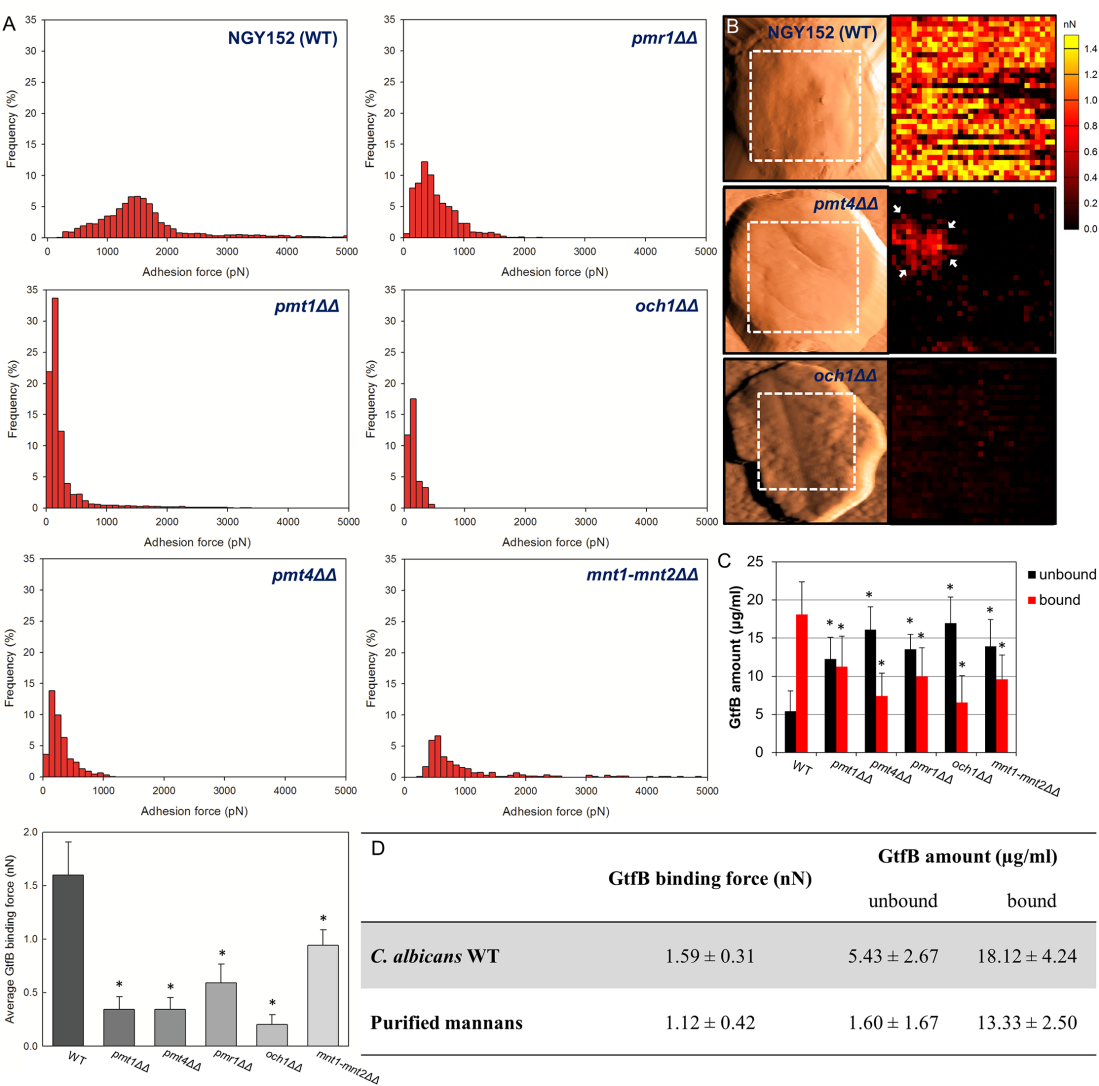
GtfB binding to *C*. *albicans* wild type and its mannosylation mutant strains. (A) Distribution and average of binding forces, (B) representative AFM scanning and force map images of wild type, *pmt4ΔΔ* and *och1ΔΔ* mutant strains (Red Hot lookup table color scheme was used to differentiate binding forces; black-to-yellow colors indicate 0–1.5 nN), (C) amount of GtfB bound on *Candida* surface, and (D) comparison of GtfB binding force between *C*. *albicans* wild type and purified mannans. The force-distance curves were obtained from at least 10 individual microbial cells from at least 3 distinct culture preparations per strain. Asterisk indicates that the values are significantly different from the *C*. *albicans* WT (*P* < 0.05).

We observed highly adhesive interactions between GtfB and *C*. *albicans* NGY152 (wild-type) or CAI4 with strong binding forces ranging from 1 to 2 nN (Fig 2 and S2A and S2B Fig). In sharp contrast, all three *O*-mannosylation mutants (*pmt1ΔΔ*, *pmt4ΔΔ*, *mnt1-mnt2ΔΔ*) showed significant reduction of GtfB binding strength. In particular, the GtfB binding forces to the surface of *pmt1ΔΔ* or *pmt4ΔΔ* (mostly <500 pN), on which the *O*-mannan was completely abrogated, were several fold less compared to wild-type strain. On the other hand, GtfB adhesive interactions to *mnt1-mnt2ΔΔ* (which has a single α mannose conserved) was slightly higher (~2.5 times) compared to the other *pmt* mutants. Interestingly, GtfB binding to *och1ΔΔ* (with defective *N*-mannan but intact *O*-mannan structure) was more severely disrupted, showing even lower binding forces (mostly <200 pN) compared to that of *pmt4ΔΔ* (S2C and S2D Fig). Although GtfB binding strength to *pmr1ΔΔ* (with partial *O*- and *N*-mannosylation defects) was reduced (200 pN-1 nN) compared to wild-type, it was significantly higher than other mannosylation mutant strains (except *mnt1-mnt2ΔΔ*).

To further detail the location of GtfB-*Candida* interactions, we used the GtfB-AFM tips to map the distribution of GtfB binding strength on the cell surface. The adhesion force maps revealed clear differences in GtfB binding profile between the wild-type and the mutant strains (Fig 2B). Large portion of the *pmt4ΔΔ* cell surface have low binding affinities, while some small areas (see arrows) show slightly higher binding forces. However, most of the *och1ΔΔ* surface was devoid or have very low GtfB binding strength. Furthermore, we also determined that GtfB binds strongly to purified mannan (~1 nN) with similar strength compared to GtfB binding to *C*. *albicans* surface (Fig 2D), while the binding force is significantly higher compared to β-glucan (zymosan) (S2E and S2F Fig).

We also determined the amount of GtfB binding on their surface to further confirm the disruption of GtfB binding to *C*. *albicans* mutant strains. Each of the *Candida* cells were incubated with GtfB (25 μg/ml) and then washed to remove unbound GtfB as detailed previously [18]. The amount of GtfB adsorbed to the cell surface, as well as that from any unbound GtfB was determined by ELISA (Fig 2C). Our data reveal significantly less GtfB bound to *C*. *albicans* mutant strains compared to their respective wild type (*P* < 0.05). Consistent with the AFM data, *pmt4ΔΔ* and *och1ΔΔ* showed the lowest amount of surface bound GtfB, which resulted in ~ 2.5 times less bound GtfB than the wild-type strains. Based on established colorimetric assays to estimate the amount of *C*. *albicans* mannoprotein [38] and carbohydrates [21, 44–47], we observed a significant reduction of both biomolecules on the mutant strains surface (S3 Fig), which correlates well with the GtfB binding data. Although defects in mannan structure caused overall reduction in GtfB binding, there are significant differences in the binding strength pattern. For example, average binding strength to *och1ΔΔ* or *pmt4ΔΔ* was 3-fold less than *mnt1-mnt2ΔΔ* and 2-fold less than *pmr1ΔΔ*. Collectively, our data show that α-mannan on the *C*. *albicans* cell surface play an important role mediating the GtfB adhesive interactions, which could affect the ability of the fungal organism to form mixed-species biofilms with *S*. *mutans*.

### *S*. *mutans-C*. *albicans* mixed-species biofilm formation

**Fig 3:**
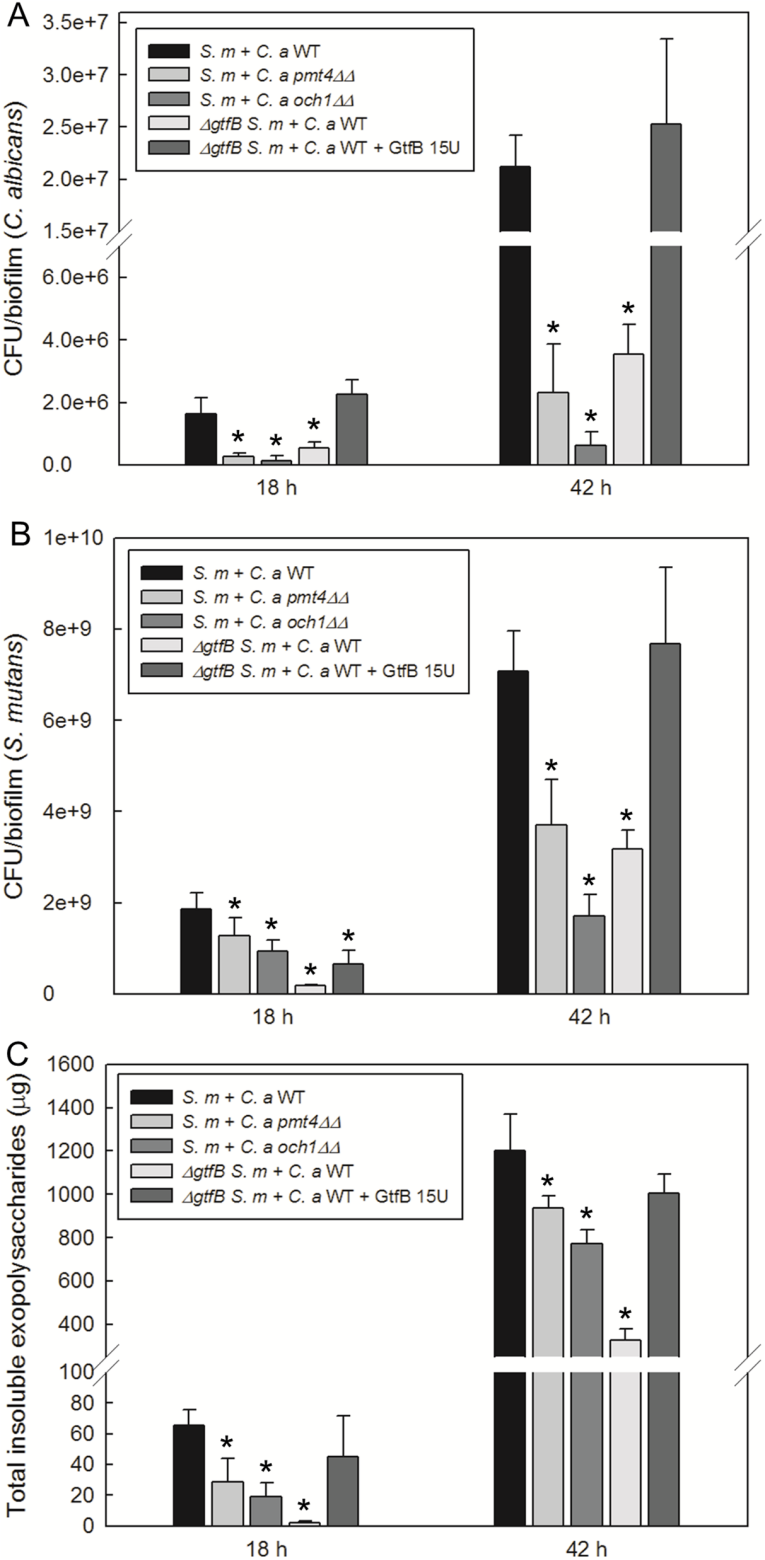
Microbiological and biochemical properties of mixed-species biofilms. (A) CFU of *C*. *albicans*, (B) CFU of *S*. *mutans*, and (C) total insoluble EPS glucan in mixed-species biofilms at early (18 h) and later (42 h) phases. Mixed-species biofilms formed with *ΔgtfB S*. *mutans* and *C*. *albicans* WT with and without GtfB supplementation (15U) were also tested. Asterisk indicates that the values are significantly different from the mixed-species biofilm formed with *S*. *mutans* and *C*. *albicans* WT (*P* < 0.05).

We examined the ability of mutant strains to form both single and mixed-species biofilms using an established sHA biofilm model [21]. Based on the biophysical and biochemical analyses, we selected the most defective GtfB binding mutant strains, *pmt4ΔΔ* and *och1ΔΔ*. Both *pmt4ΔΔ* and *och1ΔΔ* formed single-species fungal biofilms with similar number of viable cells compared to wild type strain under our experimental conditions (S4 Fig). However, we found significant differences between the mixed-species biofilms formed with *S*. *mutans* and *C*. *albicans* wild type (*S*.*m-C*.*a* WT) from those with *S*. *mutans* and *C*. *albicans och1ΔΔ* (*S*.*m-C*.*a och1ΔΔ*) or *pmt4ΔΔ* (*S*.*m-C*.*a pmt4ΔΔ*) (Fig 3). We observed significant reduction of *C*. *albicans* CFU from both *S*.*m-C*.*a och1ΔΔ* and *S*.*m-C*.*a pmt4ΔΔ* (vs. *S*.*m-C*.*a* WT) and ~ 3 times less Gtf-derived insoluble EPS glucans, while also showing disruption of *S*. *mutans* viable counts (Fig 3B and 3C). Microbiological and biochemical properties of mixed-species biofilms with all *C*. *albicans* strains are also summarized in S5 Fig. The differences between *S*.*m-C*.*a* WT and *S*.*m-C*.*a och1ΔΔ* (or *S*.*m-C*.*a pmt4ΔΔ*) were also pronounced at the later stage (42 h), particularly a substantial reduction on *C*. *albicans* viable population (> 2 log) and cell biomass as well as less insoluble glucan amounts in *S*.*m-C*.*a och1ΔΔ* (Fig 3 and S6 Fig).

**Fig 4:**
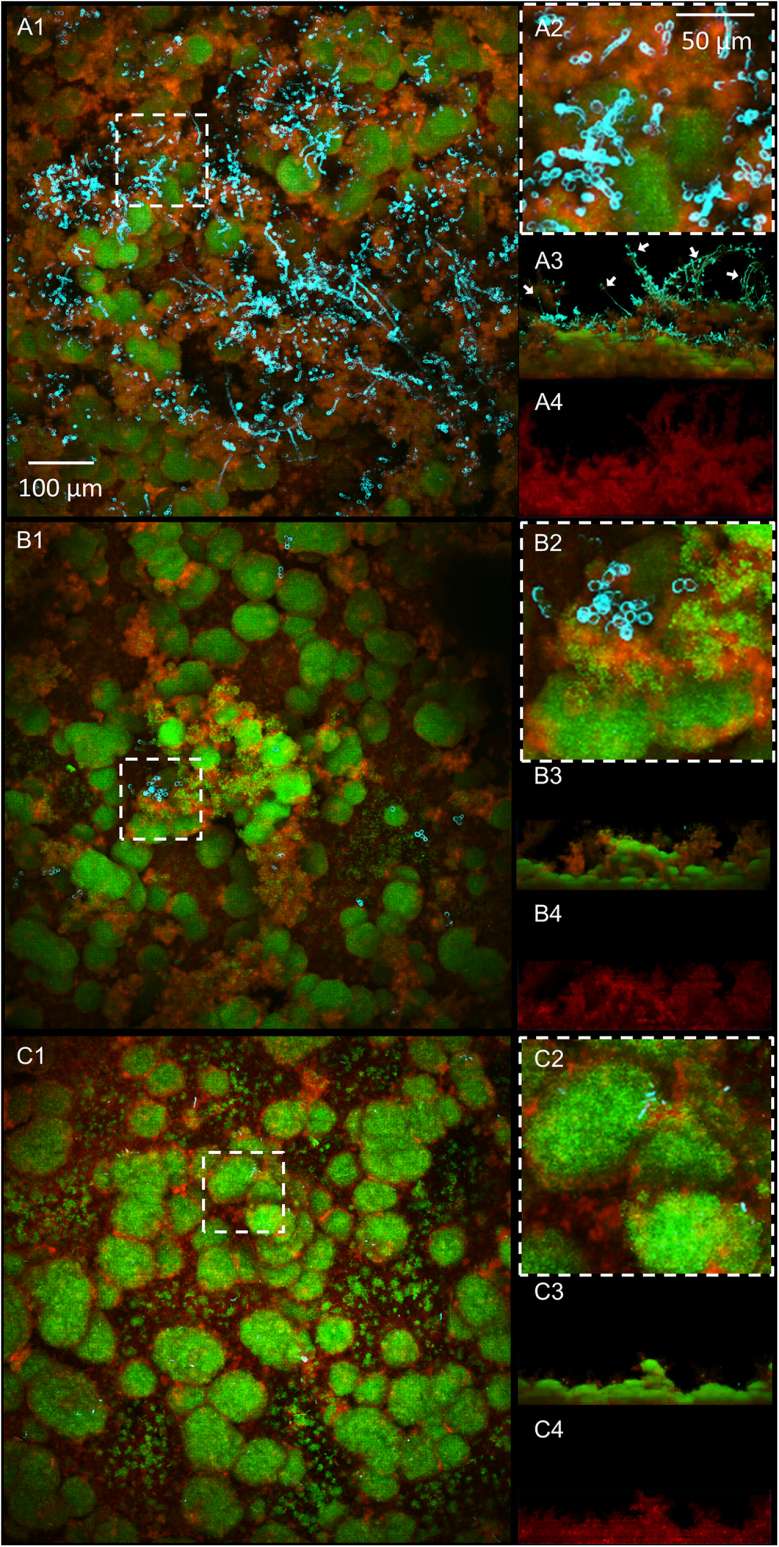
Cross-sectional and orthogonal confocal images of mixed-species biofilms. (A) *S*.*m-C*.*a* WT, (B) *S*.*m-C*.*a pmt4ΔΔ*, and (C) *S*.*m-C*.*a och1ΔΔ*. (A1-C1) Top view of biofilms, (A2-C2) magnified images of representative areas, (A3-C3) orthogonal views of biofilms, and (A4-C4) orthogonal views of EPS glucan-matrix. *S*.*m-C*.*a* WT biofilm displays numerous hyphal (mostly located in the outer layer; see white arrows) and yeast form of *C*. *albicans* with abundant amount of bacteria and densely-packed EPS glucans. In contrast, *S*.*m-C*.*a pmt4ΔΔ* and *S*.*m-C*.*a och1ΔΔ* display only a few of *C*. *albicans* with significant reduction of bacterial cells and disruption of the matrix.

Confocal images of biofilms show clear disruption of *C*. *albicans* accumulation (Fig 4A1–4C1) and α-glucan matrix (Fig 4A4–4C4) in the biofilms formed by *S*.*m-C*.*a och1ΔΔ* or *S*.*m-C*.*a pmt4ΔΔ* when compared to *S*.*m-C*.*a* WT, congruent with the microbiological and biochemical data. Although only one representative image is presented, these analyses were performed in triplicate and at least 10 images were recorded under confocal microscopy. The data is consistent with *S*. *mutans ΔgtfB* mutant strain with similarly reduced number of viable cells and Gtf-derived glucans (Fig 3) and altered architecture with fewer yeast cells. These alterations are indeed linked with a defect in GtfB-glucan synthesis, since supplementation with purified GtfB enzyme (15 U) helps to restore the mixed-species biofilm phenotype (Fig 3 and S7 Fig) in the presence of the *ΔgtfB* mutant.

Yet, it is conceivable that the defective hyphae formation and reduced growth rate of *och1ΔΔ* could influence biofilm formation, although these properties may not be key factors in mixed-species biofilm when sucrose is available. We have previously shown that *C*. *albicans* mutant strain (*efg1ΔΔ*) defective in *EFG1* (a master regulator of hyphal formation) with similar growth rate of *och1ΔΔ* developed robust mixed-species biofilms with *S*. *mutans* [21]. Thus, we conducted additional experiments with *efg1ΔΔ* as well as *bcr1ΔΔ* (key biofilm adhesion regulator), which are also known to be defective in single-species fungal biofilm formation.

**Fig 5:**
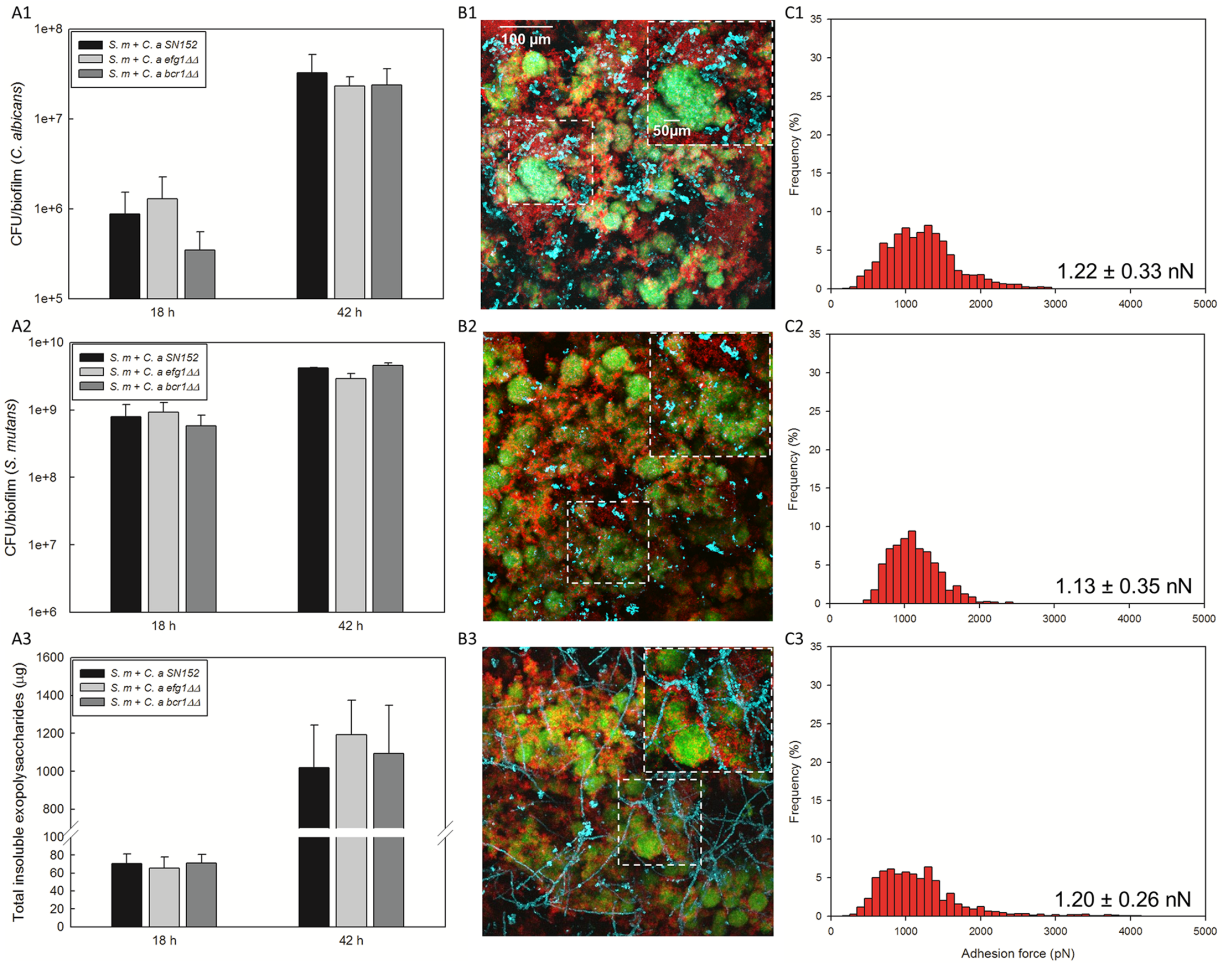
Microbiological and biochemical properties of mixed-species biofilms with other *C*. *albicans* mutant strains (*efg1ΔΔ* or *bcr1ΔΔ*) and GtfB binding strength. CFU of (A1) *C*. *albicans*, (A2) *S*. *mutans*, and (A3) total insoluble EPS in mixed-species biofilms; (B1-3) representative confocal images of mixed-species biofilms; distribution of GtfB binding forces to (C1) *C*. *a* SN152, (C2) *C*. *a efg1ΔΔ*, (C3) *C*. *a bcr1ΔΔ*, *C*. *a efg1ΔΔ* was selected due to slow growth and hyphal defect, comparable to *pmt4ΔΔ* and *och1ΔΔ*, while *bcr1ΔΔ* was selected due to lack of key biofilm adhesion regulator *BCR1* in *C*. *albicans*. In our model, reduced growth rate and hyphal defect or lack of *BCR1* of *C*. *albicans* did not affect significantly the ability of the fungal strains to develop mixed-species biofilm with *S*. *mutans* despite some morphological differences. The force-distance curves were obtained from at least 10 individual microbial cells from at least 3 distinct culture preparations per strain.

As shown in Fig 5A and 5B and S8 Fig, no significant impact on the viable population (either CFU or cell number via qPCR) and α-glucan amounts were observed when co-cultivated with *S*. *mutans* in the presence of sucrose (vs. wild-type, *P* < 0.05). Although the biofilm morphology with *och1ΔΔ* or *efg1ΔΔ* is altered due to lack of hyphal formation, their inability to form hyphae is not critical for mixed-species biofilm assembly with *S*. *mutans*. Likewise, the overall capacity to form mixed-species biofilms by *bcr1ΔΔ* was largely unaffected with noticeably more hyphal cells. Furthermore, AFM data show strong GtfB binding forces on the surface of *EFG1*- and *BCR1*-defective strains, which are similar to that between GtfB and wild-type strain (Fig 5C). Finally, it is also possible that adhesins (such as *ALS3*) under *BCR1* regulation (but potentially inducible by other transcription factors) could mediate the bacterial-fungal interaction process. Therefore, we also tested *C*. *albicans* strain (*als3ΔΔ*) defective in *ALS3* (a major adhesin associated with *S*. *gordonii* binding interaction) [43, 48]. The data show that lack of *ALS3* expression does not impair the mixed-species biofilm formation with *S*. *mutans* (S9 Fig), strongly suggesting that *ALS3* is not an essential factor in this process. Together, the data provide further support that reduced GtfB binding to mannosylation mutants play a key role in this bacterial-fungal association in the presence of sucrose.

### Mechanical stability of *S*. *mutans-C*. *albicans* mixed-species biofilm

**Fig 6:**
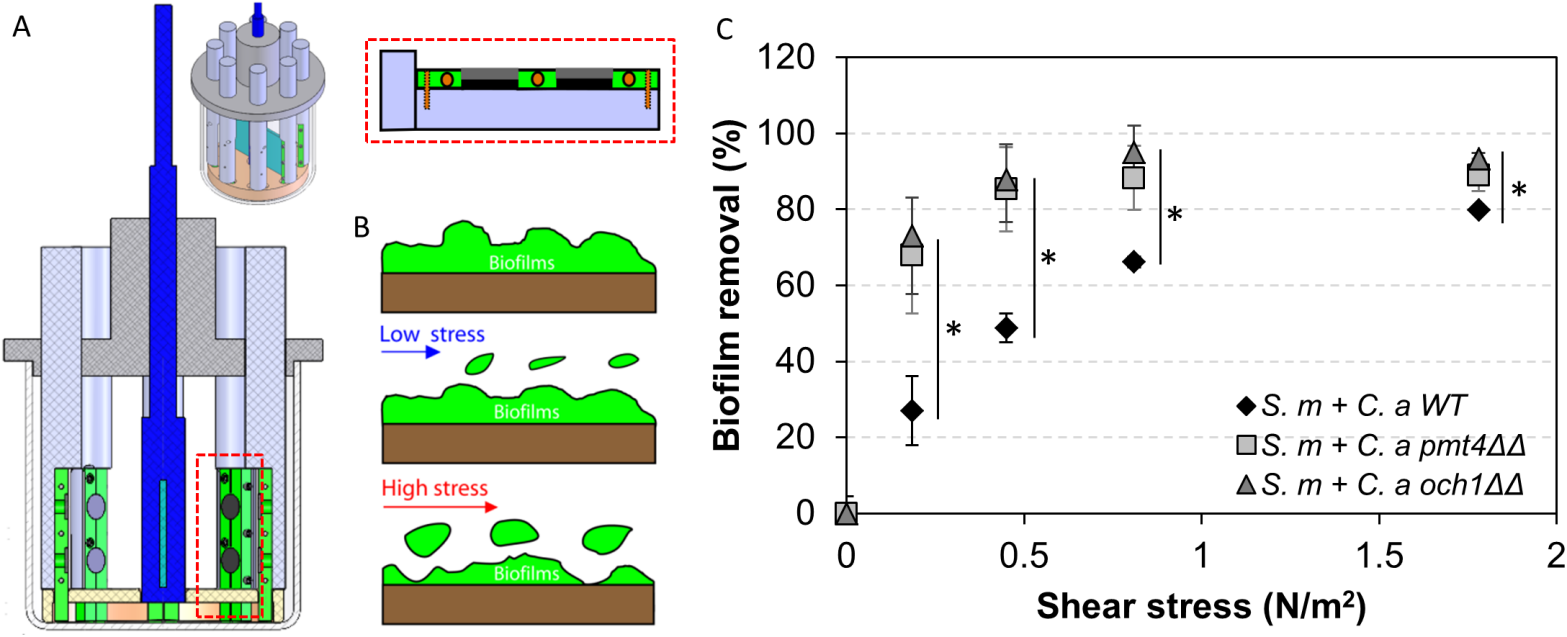
Mechanical stability of mixed-species biofilms. (A) Overview and close-up view of shear-induced biofilm mechanical strength tester, (B) schematic diagram of biofilm removal by shear stress, and (C) biofilm removal profile after application of increased shear stress. Asterisk indicates that the values are significantly different from the mixed-species biofilm formed with *S*. *mutans* and *C*. *albicans* WT (*P* < 0.05).

The biofilm cohesiveness and surface attachment strength are dependent on insoluble EPS glucans [49, 50], as they form a polymeric matrix that provides a 3D scaffold for the development of highly adherent biofilms [44]. Since the amount of insoluble α-glucan (Fig 3C) and the EPS glucan-matrix are markedly altered in *S*.*m-C*.*a och1ΔΔ* and *S*.*m-C*.*a pmt4ΔΔ* (vs. *S*.*m-C*.*a* WT), we investigated whether these structural changes could facilitate biofilm removal using a custom built device (Fig 6A and 6B) that produces shear forces to detach biofilms from the sHA surface [49].

The ability of biofilms to withstand mechanical removal under shear stress was determined by measuring the amount of biofilm that remained on the sHA before and after applying a range of shear forces. When exposed to low shear stress (0.18 N/m^2^), only a small portion of the *S*. *m-C*.*a* WT biofilm was removed (~28%), while both of *S*.*m-C*.*a och1ΔΔ* and *S*.*m-C*.*a pmt4ΔΔ* were substantially removed from sHA disc (~70%) (Fig 6C). At 0.45 N/m^2^, both of *S*.*m-C*.*a och1ΔΔ* and *S*.*m-C*.*a pmt4ΔΔ* biofilms were almost completely removed, while ~4 times stronger shear forces (1.78 N/m^2^) were required to remove equivalent amounts of *S*. *m-C*.*a* WT biofilms. It is noteworthy that hyphae can provide stability and rigidity for single-species *C*. *albicans* biofilms [51]. However, the role of hyphae in biofilm stability may be less pronounced in mixed-species biofilms, where *S*. *mutans* forms densely packed glucan-enmeshed microcolonies at the basal layers of the biofilm structure. Furthermore, in mixed-species biofilms, the hyphal forms of *C*. *albicans* are detected mostly in the outer layers of the biofilm structure (white arrows in Fig 4A3). Importantly, the amount and density of GtfB glucan-matrix (which is enhanced in the presence of *C*. *albicans* WT, but reduced in mutant strains; Fig 4A4–4C4) have been shown to be directly associated with the biofilm mechanical stability on apatitic surfaces [49]. Collectively, the data revealed that *C*. *albicans* mutants (*och1ΔΔ* and *pmt4ΔΔ*) defective in GtfB binding were compromised in forming mixed-species biofilms with *S*. *mutans*, which also weakened the mechanical stability and attachment strength, facilitating biofilm removal from sHA surface.

### Formation of plaque-biofilms on teeth *in vivo*

**Fig 7:**
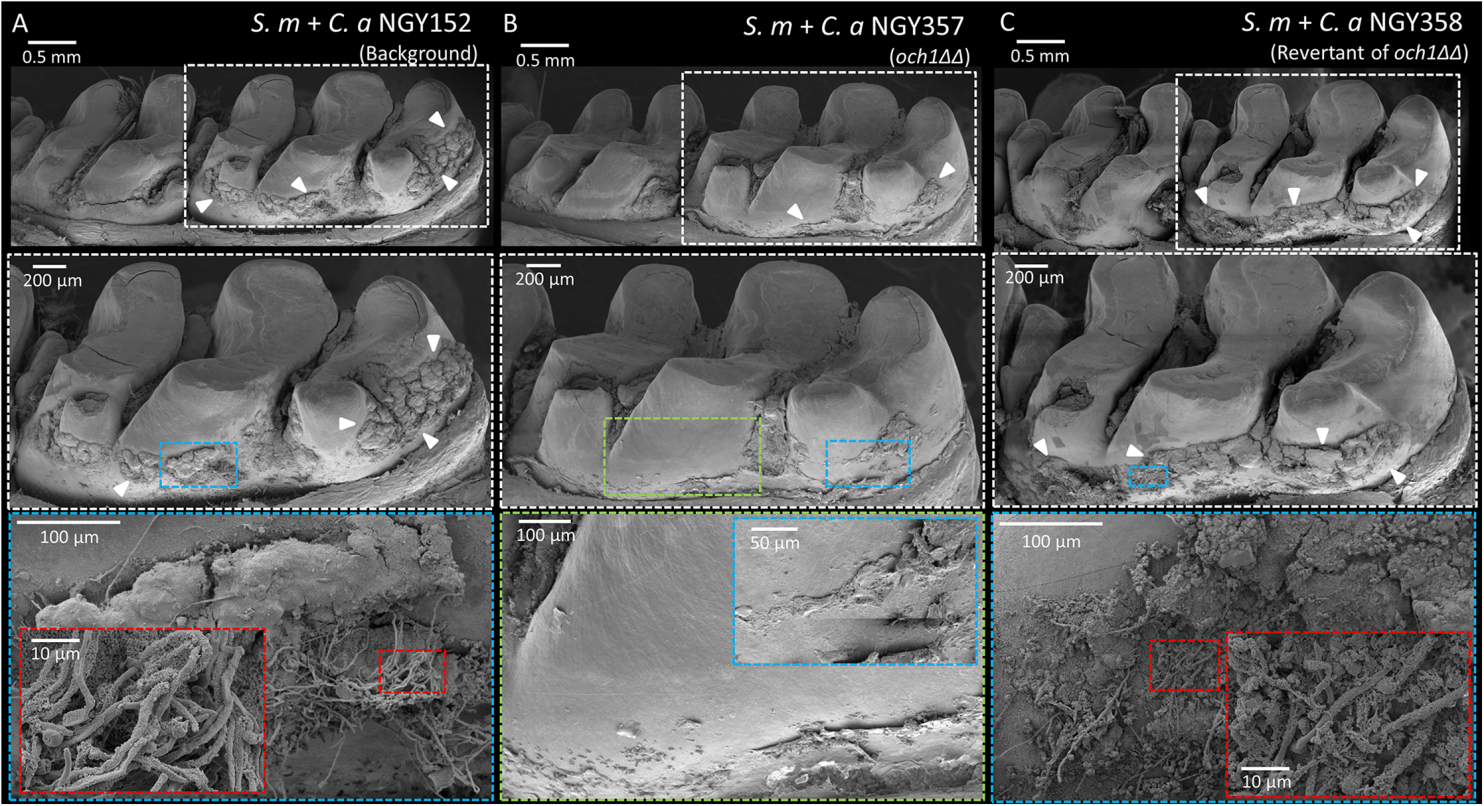
Scanning electron microscopy images of the *in vivo* plaque biofilms. Co-infected by (A) *S*. *mutans* and *C*. *albicans* WT, (B) *S*. *mutans* and *C*. *albicans och1ΔΔ*, and (C) *S*. *mutans* and revertant of *C*. *albicans och1ΔΔ*. Boxes with red dotted lines show numerous hyphal form of *C*. *albicans*, while box with blue dotted line in (B) show reduced amount of plaque without any *C*. *albicans*.

The *in vitro* data suggest that the ability of *C*. *albicans* deficient in mannoprotein to form mixed-species biofilms with *S*. *mutans* could be impaired *in vivo* when compared to wild-type strains. Thus, we sought to determine the biofilm formation by these strains using a rodent model that mimics the sugar-rich diet and microbial infection experienced clinically by children afflicted with ECC [21]. Based on *in vitro* data, we focused on *C*. *albicans och1ΔΔ*, which displayed the most defective *in vitro* biofilm. All the rats were infected with *S*. *mutans* and *C*. *albicans* (wild type strains, *och1ΔΔ* or revertant of *och1ΔΔ*) using our model, and then the impact on biofilm formation on the animal’s teeth was assessed by SEM and culturing. Co-infection with wild type strains resulted in abundant plaque-biofilm formation over the smooth surface of the teeth, containing both *S*. *mutans* and *C*. *albicans* (Fig 7A). Close-up images (Fig 7A) show hyphal forms of *C*. *albicans* populating the surface of biofilms, similar to the fungal distribution in the biofilm structure observed *in vitro*.

**Fig 8:**
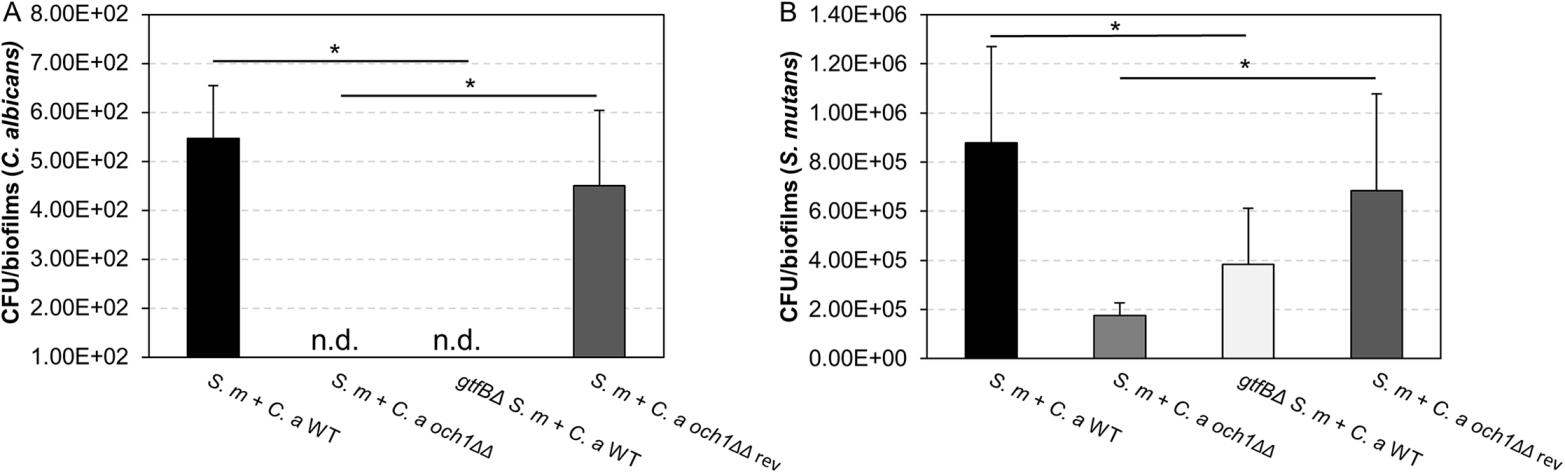
Microbiological analysis of *in vivo* plaque biofilms. CFU of (A) *C*. *albicans* and (B) *S*. *mutans*. CFU of *C*. *albicans* and *S*. *mutans* were substantially decreased when rats were coinfected by *S*. *mutans* and *C*. *albicans och1ΔΔ* or *gtfBΔ S*. *mutans* and *C*. *albicans* WT. Asterisk indicates that the values are significantly different from the mixed-species biofilm formed with *S*. *mutans* and *C*. *albicans* WT or the one with *S*. *mutans* and *C*. *albicans och1ΔΔ* revertant strain (*P* < 0.05).

In contrast, co-infection with *S*. *mutans* and *C*. *albicans och1ΔΔ* resulted in substantial reduction of the amount of plaque-biofilm on the smooth surface (Fig 7B), while fungal cells (yeast or hyphae) could not be detected. Microbiological analysis confirmed the absence of *C*. *albicans*, while the number of viable *S*. *mutans* (~5-fold less) was also significantly reduced (vs. wild type, *P* < 0.05) (Fig 8). The level of plaque-biofilm formation and the number of viable microbial cells were nearly completely recovered (vs. wild-type) when rats were coinfected with *S*. *mutans* and the revertant strain of *C*. *albicans och1ΔΔ*. Notably, coinfection of *gtfB*-defective *S*. *mutans* strain with wild-type *C*. *albicans* strain also impacted both the biofilm formation and viable population in a similar fashion of that observed with *och1ΔΔ* strain (S10 Fig), demonstrating the importance of GtfB-*Candida* interactions for their co-existence and development of cross-kingdom biofilms.

## Discussion

The results of our study indicate that mannoproteins on *C*. *albicans* surface mediate a strong and stable binding of a bacterial exoenzyme (GtfB) from *S*. *mutans*. This interaction, in turn, provides a platform through which GtfB generates EPS that is crucial for the development of a mixed species *S*. *mutans*-*C*. *albicans* plaque-biofilm *in vivo*. We have identified putative GtfB binding domains that appear to be located in the *O*- and *N*-terminal part of the mannans on *C*. *albicans* cell wall. Our data show that GtfB binds strongly to purified mannans, while demonstrating that GtfB binding strength onto *C*. *albicans* strains deficient of these outer chain mannans were severely disrupted (particularly *och1ΔΔ*), which in turn correlated with the reduced amounts of mannoproteins. Furthermore, the production of the α-glucan-matrix was significantly affected and the ability of the *Candida* strains to co-exist with *S*. *mutans* within biofilms was impaired. The data also reveal that GtfB can bind and promote mixed-species biofilms by well-characterized *C*. *albicans* mutant strains (*efg1ΔΔ* and *bcr1ΔΔ*) shown to be defective in hyphal or single-species fungal biofilm formation [3, 43, 52]. Conversely, *C*. *albicans* was unable to form mixed-species biofilms with *S*. *mutans* defective in GtfB production. It is noteworthy that GtfB can impact mixed-species biofilm with other bacterial species, but does not cause such near complete abrogation of the ability of another species (i.e. *C*. *albicans*) to co-exist with *S*. *mutans in vivo*. Collectively, our data indicate that the interactions between GtfB and mannans on the *C*. *albicans* surface play a key role for mediating this bacterium-fungus relationship found in biofilms associated with early childhood caries.

The *C*. *albicans O*-mannan is a simple linear carbohydrate comprised of a series of α1,2-linked mannose units (typically, 1–5 residues) [26]. The initial α-mannose residue is attached to the hydroxyl group of serine/threonine residues through the actions of *PMT1*, *PMT2*, *PMT4*, *PMT5* and *PMT6* [38]. Previous studies revealed that *pmt* mutant strains lack both α-mannose and a series of α1,2-linked mannose units [26]. Among five *pmt* mutant strains, *pmt1ΔΔ* and *pmt4ΔΔ* mutant strains showed drastic reduction of mannan level (at least 6-fold) on the cell wall, compared with wild type *C*. *albicans* [38], and thereby selected for this study. The abrogation of the entire α1,2-linked mannan structure greatly affected GtfB adhesive forces and distribution on the fungal surface. Interestingly, GtfB binding interactions to another *O*-mannosylation mutants, *mnt1-mnt2ΔΔ*, which is devoid of α1,2-linked mannose units but has a single α mannose left on the surface [26], was less severely disrupted compared to *pmt* mutant strains, indicating that *O*-terminal part of mannans is essential for GtfB adhesion.

The *O*-mannosylation of cell wall has been also shown to be important for the association between the commensal (viridans group) streptococci such as *S*. *gordonii* and hyphal forms of *C*. *albicans* [32] that are involved in mucosal infections [53]. However, it is important to note that *S*. *gordonii* has a completely different binding mechanism with *C*. *albicans* and it is sucrose-independent [19]. *S*. *gordonii* binds directly to the fungal surface through cell-to-cell interaction via *ALS3* expressed in hyphae [32], whereas *S*. *mutans* binds poorly to the fungal surface in the absence of sucrose [18, 19, 21, 25]. Therefore, *S*. *mutans* rely on distinctive biochemical mechanisms instead of cell-to-cell binding, whereby co-adhesion is mediated by α-glucan produced by GtfB adsorbed onto *C*. *albicans* cell wall. In this context, *ALS3* (expressed in hyphae) does not appear to be essential, as the exoenzyme binds to both yeast and hyphal cells. Furthermore, *ALS3*-defective *C*. *albicans* strain was capable of forming robust biofilms with *S*. *mutans* similar to that of wild-type. Thus, the binding mechanism of GtfB and mixed-species biofilm formation is independent of hyphae formation or the expression of major cell surface adhesins such as *ALS3*. However, it is possible that *S*. *mutans* membrane-associated glucan binding proteins (GBPs) play an important role in mediating bacterial binding to the glucan produced by GtfB adsorbed onto *Candida* surface [54].

Conversely, the core structure of *N*-mannan is a dolichol pyrophosphate anchored oligosaccharide comprised of three glucose, nine mannose and two *N*-acetylglucosamine residues (Glc_3_Man_9_GlcNAc_2_). The outer chain branched mannan is attached to the *N*-mannan core through an α1,6-backbone [26]. As a single mannosyltransferase, *OCH1*, catalyzes addition of the first α1,6-mannose, the *och1ΔΔ* mutant has no branched outer chain mannan (Fig 1) [40]. Surprisingly, adhesion forces of GtfB to *och1ΔΔ*, which has the *O*-mannan structure intact, was also substantially reduced (mostly < 200 pN), while the disruptive effect was more pronounced than that observed from *pmt1ΔΔ* or *pmt4ΔΔ* (Fig 2 and S2C and S2D Fig). This observation is consistent with biofilm experiments showing that *och1ΔΔ* is more defective in assembling EPS glucan-matrix and forming mixed-species biofilms compared to *pmt4Δ*. It is important to note that *och1ΔΔ* displays other defective phenotypes, including lack of hyphae formation and decreased growth rate. Although the morphology of *och1ΔΔ* is different due to the lack of hyphal formation, neither its inability to form hyphae nor its reduced growth rate can entirely explain its defects in mixed-species biofilm formation because other mutant strains with similar defects (e.g. *bcr1ΔΔ* and *efg1ΔΔ*) develop robust mixed-species biofilms with *S*. *mutans* in the presence of sucrose. A key concept is that GtfB binds to both yeast and hyphae, and even in absence of hyphae, mixed-species biofilms are formed due to GtfB-derived α-glucans. Nevertheless, the presence of hyphae can influence the overall architecture of the biofilms, although its role in the cariogenicity of *S*. *mutans-C*. *albicans* biofilms needs further elucidation.

Interestingly, our data also revealed that *pmr1ΔΔ* strain with shortening of both branched *N*-mannan and *O*-mannan [39] displayed the least amount of reduction in GtfB binding affinity, suggesting the importance of whole truncation of *O*- or *N*-terminal part of mannans. As higher binding forces were detected in *pmt1ΔΔ* or *pmt4ΔΔ* vs. *och1ΔΔ*, it appears that intact *N*-mannan structure is essential for adhesion stability of GtfB. Colorimetric-based assays show that reduced amount of mannans correlated with GtfB binding force data (S3 Fig). However, detailed characterization of mannan composition and content should be carried out in future studies. Furthermore, additional studies are required to elucidate the exact domains in the GtfB structure and how these motifs interact with each of the mannan components on the *Candida* surface by using GtfB with specific mutations that produce EPS glucans but incapable of binding to mannan or *C*. *albicans* mutant strains overexpressing mannans.

Based on protein chemistry and biochemical analyses, a series of repeat motifs of amino acids found at the carboxy terminus of Gtfs (glucan-binding domain) could recognize and bind to glucose moieties [22, 55]. However, recent elucidation of Gtf crystal structure combined with 3D modelling revealed several additional binding motifs [56–59]. Moreover, the Gtf protein-ligand mechanisms are rather complex, involving intricate conformational changes (e.g. decrease in helical and increase in β-sheet structures) when adsorbing to solid or semi-solid surfaces [56]. Interestingly, GtfC binds less effectively than GtfB, while GtfD does not bind to *Candida* surface [18]. Thus, it is conceivable that multiple yet specific sites in the GtfB structure may be interacting with mannans to allow optimal binding and stability on *C*. *albicans* surface. We found that GtfB binds with higher binding strength to mannans compared to β-glucan (zymosan) (Suppoting Information S2E and S2F Fig), indicating some selectivity to a specific carbohydrate moiety or glycosydic linkage. Further studies using *in situ* real-time spectroscopy shall elucidate the binding domains and the conformational changes the enzyme undergoes during GtfB-mannan interactions. Nevertheless, it is clear that disruption of both *O*- and *N*-mannan is required to effectively thwart this unique bacterial-fungal interaction.

At the same time, we recognize the interactions between these two organisms are complex [60] and could possibly induce additional responses in one another and alter the surrounding biofilm microenvironment. For example, further downstream analysis of post-GtfB binding effects may reveal additional molecular mechanisms that could trigger responses by *C*. *albicans* at single-cell level, which can modify the biofilm composition and structure as a whole [61]. The possibility of combining biophysical methods with high-resolution microscopy for simultaneous spatio-temporal analysis of microenvironment and gene expression [62] could facilitate such detailed characterization. Moreover, this unique GtfB-mannan coupling mechanism also helps the fungus to colonize teeth (not their normal habitat). Thus, enhanced colonization by *C*. *albicans* and increased carriage in mixed-species biofilms on teeth may also provide a fungal reservoir that could promote fungal infection of oral mucosal surfaces [21, 63]. Further *in vivo* studies shall determine the impact of mannan-GtfB interaction on the severity of tooth decay as well as on *Candida* infection of oral soft tissues.

In summary, we have found that *C*. *albicans* strains lacking genes critical for the biosynthesis of either *N*- or *O*-linked mannans showed severely reduced GtfB binding relative to wild type strains. Furthermore, these mutant strains were significantly impaired in their ability to form mixed-species biofilms with *S*. *mutans* in the presence of sucrose, showing substantial reduction in both the EPS α-glucan content and microbial population. Notably, the mechanical stability of the defective biofilm was significantly attenuated, resulting in near complete biofilm removal by shear forces. Altogether, the data reveal a key role for *C*. *albicans O*-mannan and *N*-mannan outer chain in mediating GtfB binding, glucan-matrix assembly and mixed-species biofilm development *in vivo*. The unveiled mechanism emphasizes the need to target *C*. *albicans* either by blocking the binding of Gtfs to the fungal cell wall or use of topical antifungal agents. Enhanced understanding of GtfB-*Candida* interactions may accelerate progress towards devising new and effective therapies to disrupt this cross-kingdom biofilm associated with an important childhood oral disease.

## Materials and methods

### Microorganisms and culture conditions

*Candida albicans* NGY152 or CAI4 and its mannosylation mutant (and revertant) strains were used for GtfB binding and biofilms assays (gift from Neil Gow, University of Aberdeen) (Table 1). *Streptococcus mutans* UA159 serotype c (ATCC 700610), a virulent cariogenic pathogen and well-characterized EPS producer, was used for mixed-species biofilm experiments. The cultures were stored at −80°C in tryptic soy broth (*S*. *mutans*) or Sabouraud dextrose broth (*C*. *albicans*) containing 20% glycerol. All strains of *C*. *albicans* and *S*. *mutans* cells were grown to mid-exponential phase (optical densities at 600 nm of 0.6) in ultrafiltered (10-kDa molecular-mass cutoff; (UFTYE; pH 5.5) containing 1% (wt/vol) glucose and harvested by centrifugation (6,000 × g, 10 min, 4°C) as described elsewhere [18]. Then, the cells were washed 3 times in phosphate-buffered saline (pH 7.0 to 7.2; HyClone Laboratories Inc., Logan, UT, USA) before cell immobilization.

### AFM methodology

Schematic diagram of single-molecule AFM analysis is shown in S1 Fig. Prior to AFM analysis, microbial cells were immobilized on a poly-L-lysine-coated glass slide [23, 64]. Briefly, a glass slide was prepared by deposition of poly-L-lysine solution (0.1% w/v in H_2_O; Sigma-Aldrich, St. Louis, MO, USA) by overnight incubation. Washed cells were immobilized on a positively charged poly-L-lysine coated glass slide for 1 h at room temperature. Then, the glass slide was gently washed with demineralized water to remove loosely adhered cells, and the slide was kept hydrated prior to AFM analysis. The immobilized cells were verified for viability using BacLight Live/Dead, as detailed elsewhere [23]. Also, we tested the GtfB binding to commercially available purified mannan (Sigma-Aldrich M7504) and zymosan A (Sigma-Aldrich Z4250) as detailed elsewhere [21]. Briefly, glass slide was modified with 10% DVS in PBS (pH 7.4) at room temperature overnight with gentle rotation, followed by rinsing 3 times with PBS. Then, 2.5mg of mannan or zymosan A was dissolved in 1ml of coupling buffer (0.1M NaHCO_3_, pH around 8.1) containing 5% PEG. The mixture was added to DVS modified glass slide and incubated overnight at room temperature with gentle rotation. Then, the glass slide was washed 3 times with PBS to remove unbound ligand. The remaining unreacted DVS sites were blocked by addition of ethanolamine (1M pH 9) for 30min at room temperature with gentle rotation. Finally, the functionalized glass slides were kept under wet condition at 4°C for AFM measurement.

The GtfB enzyme was prepared and purified to near homogeneity via hydroxyapatite column chromatography as detailed previously [18]. To functionalize the AFM tips with GtfB, the cantilever tips were cleaned by immersing them in nitric acid for 5 min, followed by washing them in demineralized water 3 times. Then, AFM tips were exposed to saturating amounts of GtfB for 1 h at room temperature [18]. The GtfB functionalization was verified using our monoclonal antibody to GtfB and fluorescence imaging via Alexa Fluor-labeled goat anti-mouse IgG secondary antibody (see S1 Fig). All force measurements were conducted in fluid-phase at room temperature under phosphate-buffered saline (HyClone Laboratories Inc.) using an MFP-3D AFM (Asylum Research, Santa Barbara, CA, USA) as detailed elsewhere [23]. Briefly, silicon nitride probes (TR400PSA, Olympus, Tokyo, Japan) with a resonance frequency of ~11 kHz and spring constant of ~0.02 N/m and a nominal tip radius of 20 nm were used. Cells immobilized on glass surfaces were imaged in contact mode at randomly selected locations at 1 Hz, and an adhesion force map was obtained. Cantilever deflection data upon retraction from cell surfaces were acquired and converted to force data. The force-distance curves were obtained from at least 10 individual microbial cells from at least 3 distinct culture preparations per strain.

### Gtfs binding to the *C*. *albicans* cell surface or purified mannans

The Gtfs were adsorbed to the microbial cells as detailed elsewhere [18] and bound GtfB was determined by Enzyme-Linked Immunosorbent Assay (ELISA) and anti-GtfB monoclonal antibody as described elsewhere [65, 66] with some modifications. Briefly, *C*. *albicans* yeast cells (ranging from 1 × 10^7^ to 2 × 10^7^/ml) were mixed with saturating amounts of GtfB (25 μg/ml) in adsorption buffer and incubated for 60 min at 37°C as optimized previously [18]. Then, the cells were pelleted by centrifugation (6,000 × g, 10 min. 4°C) and the supernatant was saved. The pellet was then resuspended (washed) with adsorption buffer and centrifuged, and the supernatant collected. The washing step was repeated twice and the all the supernatants (containing unbound GtfB) were saved; the cell pellet was resuspended in the same final volume of the combined supernatant (1 ml). Subsequently, an aliquot of 100 μl of washed cells (with adsorbed GtfB) or the supernatant (unbound) were added to the 96-well and incubated overnight at 4 ^o^C; cell and supernatant without GtfB treatment were used as background control. Then, the solution was discarded and each well was washed with PBST (0.05% Tween 20 in 1X PBS) three times (200 μl/well, 5min) with rocking, and 200 μl of 1% BSA (Sigma-Aldrich, Saint Louis, MO) in PBS was added and incubated at 37°C for 1hr. Each well was washed with PBST three times, and anti-GtfB monoclonal antibody (GtfB mAb) was added to each well, and incubated at 37°C for 2 hr. Following the washing procedure with PBST, each well was incubated with secondary antibody (goat anti-mouse IgG-HRP; Life technology, Catalog #: 626520) at the ratio of 1:2000 with 1% BSA at 37°C for 1 hr. After PBST washing, the amount of GtfB was determined by colorimetric assay using 3,3,5,5-tetramethylbenzidine (TMB; 10 mg/ml, 10 mg TMB dissolved in 1 ml DMSO) and H_2_O_2_ using a standard curve prepared from 1 μg/ml of GtfB. Absorbance was then read at 450 nm on a microplate reader (Genios Pro, Tecan, Durham, NC). We also tested GtfB binding to mannans by using commercially available purified mannans (Sigma-Aldrich M7504) as detailed elsewhere [21]. Briefly, we coupled purified mannans to DVS-magnetic beads following the manufacturer’s protocol (Bioclone, Inc). After ligand coupling, the ligand-coated beads were mixed with saturating amount of GtfB (25 μg/ml), and we followed the procedures described above. All assays were done in quadruplicate in at least three different experiments.

### Quantification of mannoproteins and carbohydrates on *C*. *albicans* cell surface

Amount of mannoprotein on *Candida* surface was analyzed as detailed elsewhere [38]. Briefly, each strain was cultured overnight in 500 ml of LMW medium supplemented with 1% glucose at 30°C to an OD_600_ nm of about 1.5. Cells were harvested, and washed extensively with 1M NaCl and 1mM PMSF as described previously [67]. Then, washed cells were lyophilized, followed by extraction using 1M NaOH for 30 min at 100°C. The amount of mannoprotein was measured and quantified via Folin’s reagent by using bovine serum albumin as the standard curve. Quantification of total carbohydrate was performed using established colorimetric methods [21, 44–47]. At least 3 independent experiments were performed for each of the assays.

### *In vitro* biofilm model

Biofilms were formed using our saliva-coated hydroxyapatite (sHA) model as described previously [21, 44, 68]. Briefly, the hydroxyapatite discs (surface area, 2.7 ± 0.2 cm^2^; Clarkson Chromatography Products, Inc., South Williamsport, PA) coated with filter-sterilized, clarified whole saliva (collected by chewing paraffin wax from 3 individuals) were vertically suspended in 24-well plates using a custom-made wire disc holder, mimicking the free smooth surfaces of the pellicle-coated teeth [44, 68]. Each disc was inoculated with approximately 2 × 10^6^ CFU of *S*. *mutans*/ml and 2 × 10^4^ CFU of *C*. *albicans*/ml (containing predominantly yeast cell forms [18]) in ultrafiltered UFYTE broth containing 1% (30 mM) sucrose at 37°C under 5% CO_2_; the proportion of the microorganisms in the inoculum is similar to that found in saliva samples from children with ECC [13, 21]. The culture medium was changed twice daily at 8 am and 6 pm until the end of the experimental period (42 h). The biofilms were collected at 18 h and 42 h based on the developmental stage of the mixed-species biofilms using the sHA model [21].

### Quantitative biofilm analysis

The development of each of the biofilms was assessed at each time-point using our well-established protocols optimized for biofilm imaging and quantification [44, 68]. We examined the 3D architecture and the spatial distribution of Gtf-derived EPS glucans and microbial cells within intact biofilms. Briefly, EPS glucans were labelled via incorporation of Alexa Fluor 647 dextran conjugate (Molecular Probes Inc., Eugene, OR) during biofilm formation. The labelling method is highly specific for GtfB-derived α-glucan because the fluorescently labelled dextran serves as a primer for GtfB and is directly incorporated into glucans during EPS matrix synthesis over the course of biofilm development [57, 64]. This method cannot label *C*. *albicans* derived β-glucans as demonstrated previously [21] and further illustrated in S11 Fig. *S*. *mutans* was stained with Syto 9 (485/498 nm; Molecular Probes), while *C*. *albicans* was labeled with ConA-tetramethylrhodamine (Molecular Probes) as described previously [21]. Imaging was performed using a multi-photon laser scanning microscope (SP5, Leica Microsystems, Buffalo Grove, IL,USA) equipped with a 20 X (1.0 numerical aperture) water immersion lens. Each biofilm was scanned at 5 positions randomly selected on the microscope stage, and confocal image series were generated by optical sectioning at each of these positions. Furthermore, a separate set of biofilms was used for standard microbiological and biochemical analysis. The biofilms were removed and homogenized by sonication, and the number of viable cells (total number of CFU per biofilm) was determined [45]. It is noteworthy that CFU data for *C*. *albicans* have limitations given the morphology, since the cells exist in both the yeast and hyphal forms; the latter are essentially multicellular structures that, when plated, form a single CFU, despite having a larger biomass than yeast forms. However, it is noteworthy that, despite limitation, CFU counting has been the standard technique to enumerate the *C*. *albicans* viable cells. We have also conducted computational analysis using COMSTAT to determine the total biomass of *C*. *albicans* to complement the CFU analysis (S6 Fig). Quantification of polysaccharides was performed using established colorimetric methods as detailed previously [21, 44–47]. At least 3 independent biofilm experiments were performed for each of the assays.

### Isolation of DNA and quantification of *C*. *albicans* population via quantitative real time PCR (qPCR)

Genomic DNA of *C*. *albicans* was isolated using MasterPure DNA purification kit (Epicentre, Madison, WI, USA) as described previously [47]. Briefly, harvested biofilms at 18 h or 42 h were resuspended with 100 μL of TE (50 mM Tris, 10 mM EDTA, pH 8) and incubated with 10.9 μL lysozyme (100 mg/mL stock) and 5 μL mutanolysin (5U/μL stock) at 37°C and 30°C respectively. Genomic DNA was then isolated by using the DNA purification kit. One nanogram of genomic DNA per sample was amplified with a Bio-Rad CFX96 using SYBR green supermix (Bio-Rad Laboratories, Inc., Hercules, CA, USA) and *C*. *albicans* specific primer: Forward 5ʹ-AGAACGATAATAACGACGATGA-3ʹ and reverse 5ʹ-AGTCATTGTAGTAATCCATCTCA-3ʹ. A standard curve based on the genomic size of *C*. *albicans* (15.6 Mb) and one genome copy represents one *C*. *albicans* cell. The standard curve (range tested was 10^7^ to 10^2^ cells (PCR efficiency was 97% with *r*^2^ = 0.995)) was used to transform the quantification cycle (Cq) values to the relative number of *C*. *albicans*. To determine the number of *C*. *albicans* in the original sample, the number of cells detected in the qPCR runs was multiplied by the dilution factor from the DNA dilution step.

### Analysis of the mechanical stability of biofilms

The mechanical stability of mixed-species biofilms was compared using a custom built device [49]. Biofilms formed on sHA were placed in the disk holder of the device (Fig 6A), and then exposed to constant shear stress of 0.18, 0.45, 0.81 and 1.78 N/m^2^ for 10 min. The duration of 10 min of shearing was determined to have reached a steady state of biofilm removal based on our previous study [49]. The amount of remained biofilm dry-weight (biomass) before and after application of shear stress for each condition was determined. Then, the percentage of biofilm removed from the sHA disc surface was calculated as described previously [49].

### *In vivo* model of dental plaque-biofilm

Animal experiments were performed using an established animal (rat) caries model [21, 47, 69]. Briefly, six litters of 8 female Sprague Dawley rats aged 15 days were purchased with their dams from Harlan Laboratories (Madison, WI). Upon arrival, animals were screened for *S*. *mutans* and *C*. *albicans*, and were determined not to be infected with either organism, by plating oral swabs on selective media: ChromAgar (VWR International LLC, Radnor, PA) for *C*. *albicans* and Mitis Salivarius Agar plus Bacitracin (MSB) for *S*. *mutans*. The animals were then infected by mouth with actively growing culture of *S*. *mutans* UA159 and each of the *C*. *albicans* wild-type or mutant strains (see below) by 23 days as detailed previously [21]. All the animals were randomly placed into one of the following four groups: (1) *S*. *mutans* UA159 plus *C*. *albicans* NGY152 infected, (2) *S*. *mutans* UA159 plus *C*. *albicans och1ΔΔ* infected, (3) *S*. *mutans* UA159 plus revertant of *C*. *albicans och1ΔΔ* infected, and (4) *gtfBΔ S*. *mutans* plus *C*. *albicans* NGY152 infected. Animals were screened at 26, 28, and 30 days for *S*. *mutans* and *C*. *albicans* infection. Each of the infected groups was confirmed for its respective microbial infection using colony quantitative real-time PCR; no cross-contamination was observed throughout the experiment. All animals were provided the National Institutes of Health cariogenic diet 2000 and 5% sucrose water *ad libitum*. The experiment proceeded for 3 weeks. At the end of 3 weeks, the animals were sacrificed. The jaws were aseptically dissected and were processed for microbiological analysis of each animal’s plaque biofilms as described elsewhere [47]. For microbiological analysis, the left jaws were sonicated in 5 ml of 154 mM sterile NaCl solution for plaque biofilm removal. The suspensions obtained were serially diluted and were plated on MSB or ChromAgar to estimate the *S*. *mutans* or *C*. *albicans* population, respectively, and on blood agar to determine the total cultivable flora in the plaque biofilms. Plaque biofilms were also characterized using scanning electron microscopy (SEM; Quanta FEG 250, FEI, Hillsboro, OR) at the Electron Microscopy Resource Laboratory at the University of Pennsylvania.

### Ethics statement

The animal experiment was performed in strict accordance with the guidelines of the Animal Welfare Act of the United States, under the protocol reviewed and approved by the Institutional Animal Care and Use Committee of the University of Pennsylvania (IACUC#805735). The whole saliva is a convenient sample (with no identifiers) collected for the sole purpose of coating the hydroxyapatite discs for the *in vitro* biofilm studies. All adult subjects provided written informed consent (no children participated in the saliva collection) under the protocol reviewed and approved by the University of Pennsylvania Research Subject committee (IRB#818549).

### Statistical analyses

The data were analyzed by pairwise comparisons of multiple groups with regression models using ranked values. Kruskal–Wallis tests, which are non-parametric and based on ranks, were used for two-group comparisons. The significance levels were set at 5%, and no adjustments were made for multiple comparisons. SAS statistical software, version 9.3 (SAS Institute, Cary, NC), was used to perform the analyses.

## Supporting information

S1 Fig Single-molecule atomic force microscopy (AFM). (A) Schematic diagram of single-molecule AFM, (B) fluorescent image of AFM tip without GtfB functionalization, (C) fluorescent image of GtfB-functionalized AFM tip; Alexa 488–labeled monoclonal antibody (goat anti-mouse IgG [H+L]–HRP) was bound to GtfB on the AFM tips to verify functionalization, (D) AFM force measurement.
(TIF)

S2 Fig GtfB binding forces to (A) *C*. *albicans* CAI4, (B) *C*. *albicans* NGY152, (C) *C*. *albicans pmt4ΔΔ*, (D) *C*. *albicans och1ΔΔ* strains, (E) purified mannans, and (F) purified β-glucans. The force-distance curves were obtained from at least 10 individual microbial cells from at least 3 distinct culture preparations per strain. Purified mannans and β-glucans were tested in quadruplicate.
(TIF)

S3 Fig Amounts of total mannoproteins and carbohydrates on *C*. *albicans* surface. Asterisk indicates that the values are significantly different from each other (*P* < 0.05).
(TIF)

S4 Fig The number of viable cells in single-species biofilm formed by each of *C*. *albicans* wild-type, *pmt4ΔΔ*, and *och1ΔΔ* strain at early (18 h) and later (42 h) phases. (TIF)

S5 Fig Microbiological and biochemical properties of mixed-species biofilms with wild-type and all mutant strains of *C*. *albicans* tested in this study. CFU of (A) *C*. *albicans*, (B) *S*. *mutans*, and (C) total insoluble EPS in mixed-species biofilms. Asterisk indicates that the values are significantly different from the mixed-species biofilm formed with *S*. *mutans* and *C*. *albicans* WT (*P* < 0.05).
(TIF)

S6 Fig COMSTAT analysis of biovolume of *C*. *albicans* in mixed-species biofilms. Asterisk indicates that the values are significantly different from the mixed-species biofilm formed with *S*. *mutans* and *C*. *albicans* WT or the one with *ΔgtfB S*. *mutans* and *C*. *albicans* WT supplemented with 15 U of GtfB (*P* < 0.05).
(TIF)

S7 Fig Representative confocal images of mixed-species biofilms formed with *ΔgtfB S*. *mutans* and *C*. *albicans* WT. (A1) Top view and (A2) orthogonal views of biofilms with no GtfB supplemented; (B1) top view and (B2) orthogonal views of biofilms with 15 U of GtfB supplemented.
(TIF)

S8 Fig Comparison of *C*. *albicans* populations between CFU and cell number via qPCR methods. There are no significant differences in both CFU and cell number between *C*. *albicans* wild type and mutant strains.
(TIF)

S9 Fig Microbiological and biochemical properties of mixed-species biofilms with *C*. *albicans* mutant strain defective in *ALS3* (*als3ΔΔ*). CFU of (A) *C*. *albicans*, (B) *S*. *mutans*, and (C) total insoluble EPS in mixed-species biofilms. In our model, *C*. *albicans* defective in *ALS3* expression was capable of developing robust mixed-species biofilm with *S*. *mutans*.
(TIF)

S10 Fig Representative SEM images of the *in vivo* plaque biofilms from the rat coinfected by *gtfBΔ S*. *mutans* and *C*. *albicans* wild-type. (TIF)

S11 Fig Confocal images of *C*. *albicans* single-species biofilms with Alexa Fluor 647 dextran-conjugate labeling with or without GtfB supplementation. (A) *C*. *albicans* single-species biofilm showed no labeling of fungal β-glucan in the absence of GtfB, (B) while abundant labelled glucan (in red) was observed when GtfB (15 U) was added during *C*. *albicans* biofilm formation, demonstrating highly specific labeling of α-glucans produced by GtfB.
(TIF)
