# Supplementary figures and images for: Candida albicans mannans mediate Streptococcus mutans exoenzyme GtfB binding to modulate cross-kingdom biofilm development in vivo
Source: PLoS Pathog. 2017 Jun 15;13(6):e1006407. doi: 10.1371/journal.ppat.1006407 (PMC5472321; doi:10.1371/journal.ppat.1006407)

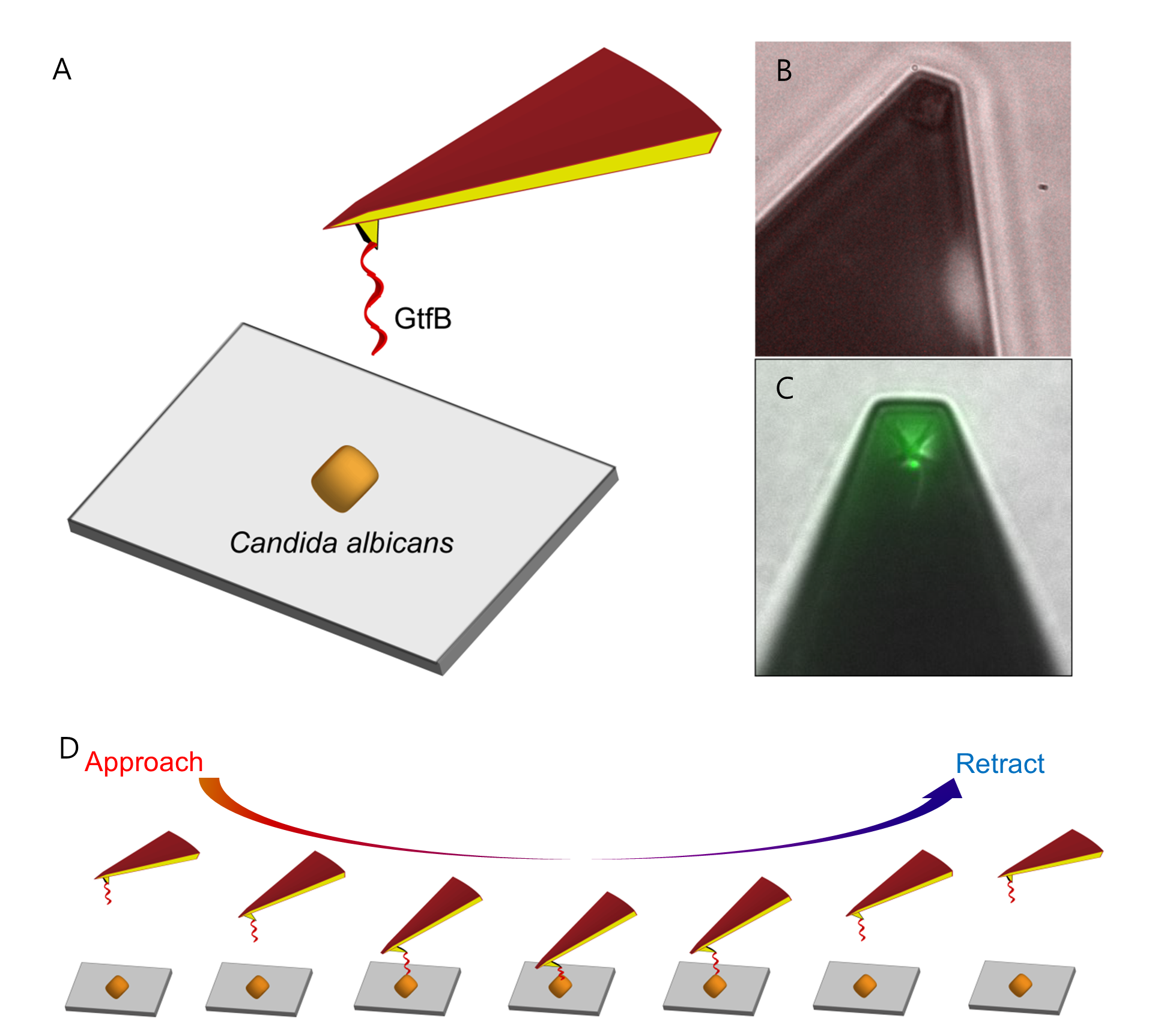

Supplement: S1 Fig — (A) Schematic diagram of single-molecule AFM, (B) fluorescent image of AFM tip without GtfB functionalization, (C) fluorescent image of GtfB-functionalized AFM tip; Alexa 488–labeled monoclonal antibody (goat anti-mouse IgG [H+L]–HRP) was bound to GtfB on the AFM tips to verify functionalization, (D) AFM force measurement. (TIF) [file ppat.1006407.s001.tif]

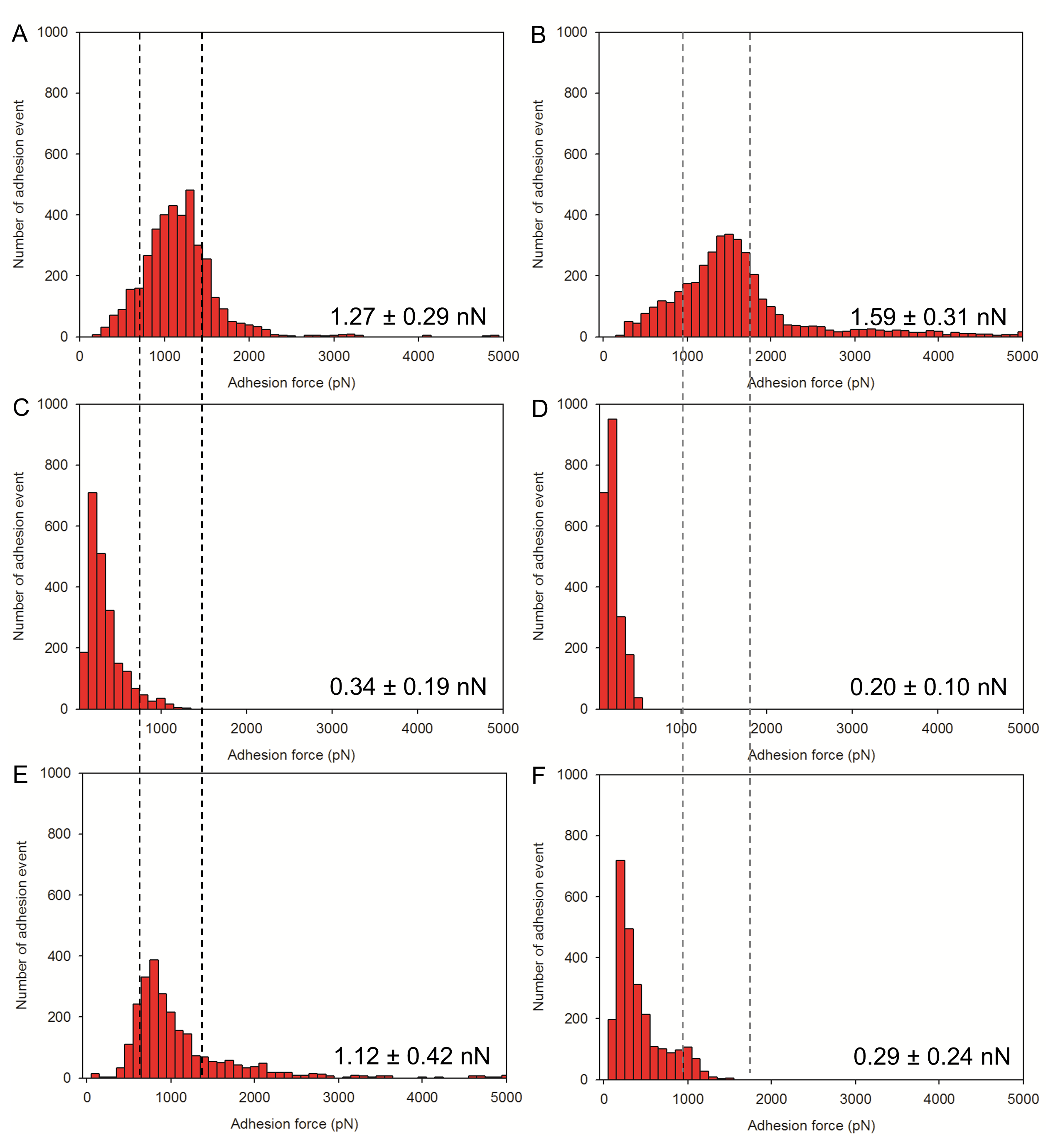

Supplement: S2 Fig — GtfB binding forces to (A) C. albicans CAI4, (B) C. albicans NGY152, (C) C. albicans pmt4ΔΔ, (D) C. albicans och1ΔΔ strains, (E) purified mannans, and (F) purified β-glucans. The force-distance curves were obtained from at least 10 individual microbial cells from at least 3 distinct culture preparations per strain. Purified mannans and β-glucans were tested in quadruplicate. (TIF) [file ppat.1006407.s002.tif]

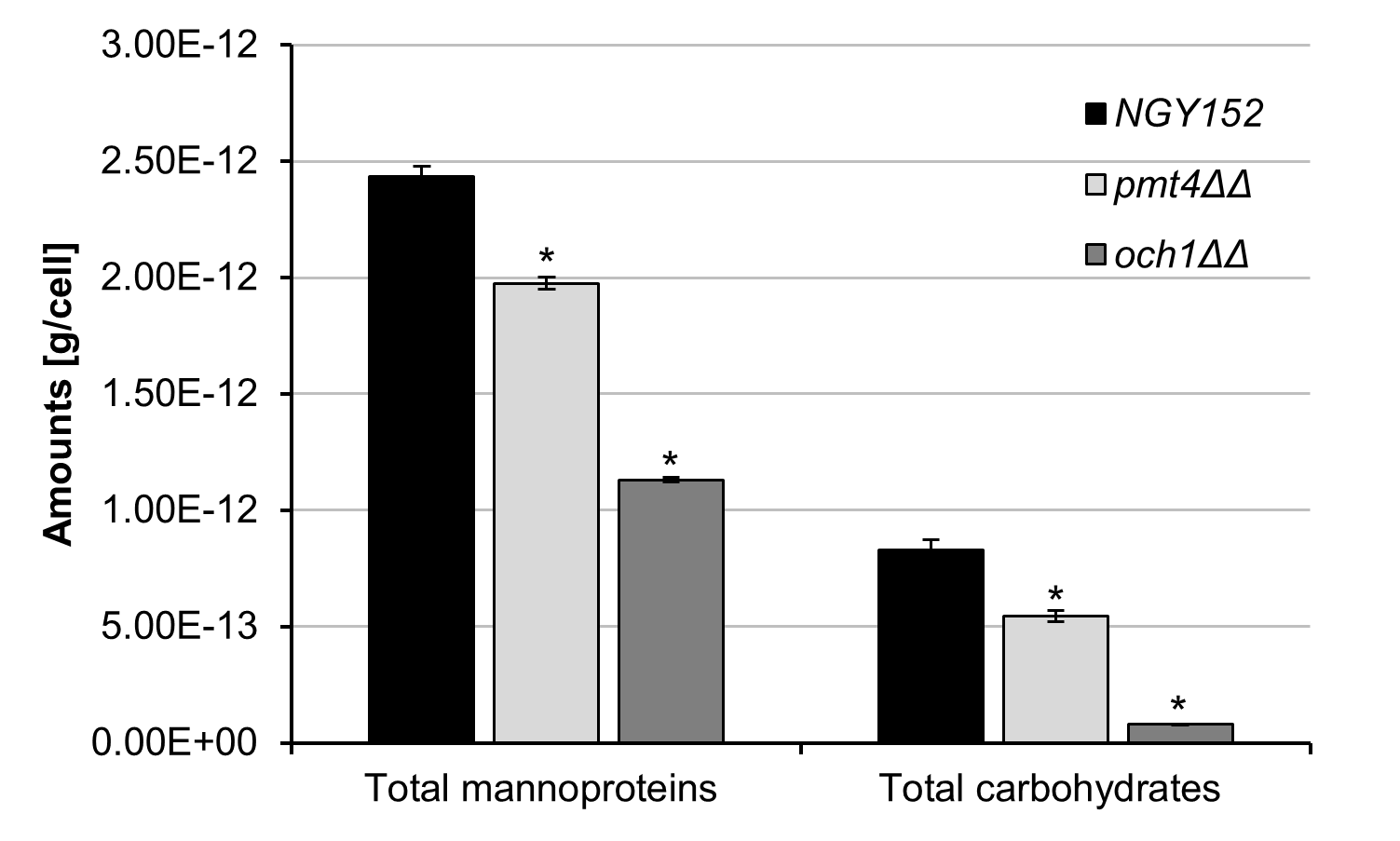

Supplement: S3 Fig — Asterisk indicates that the values are significantly different from each other (P < 0.05). (TIF) [file ppat.1006407.s003.tif]

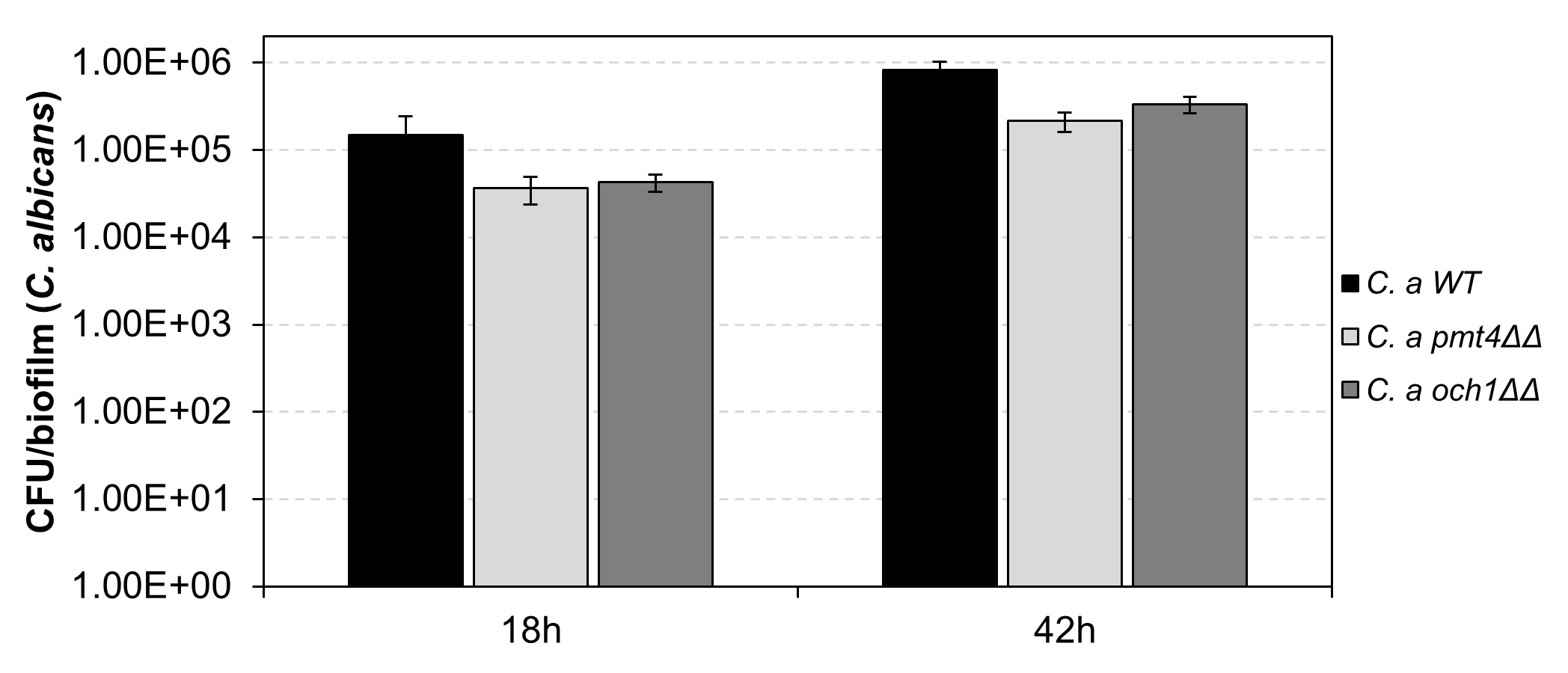

Supplement: S4 Fig — (TIF) [file ppat.1006407.s004.tif]

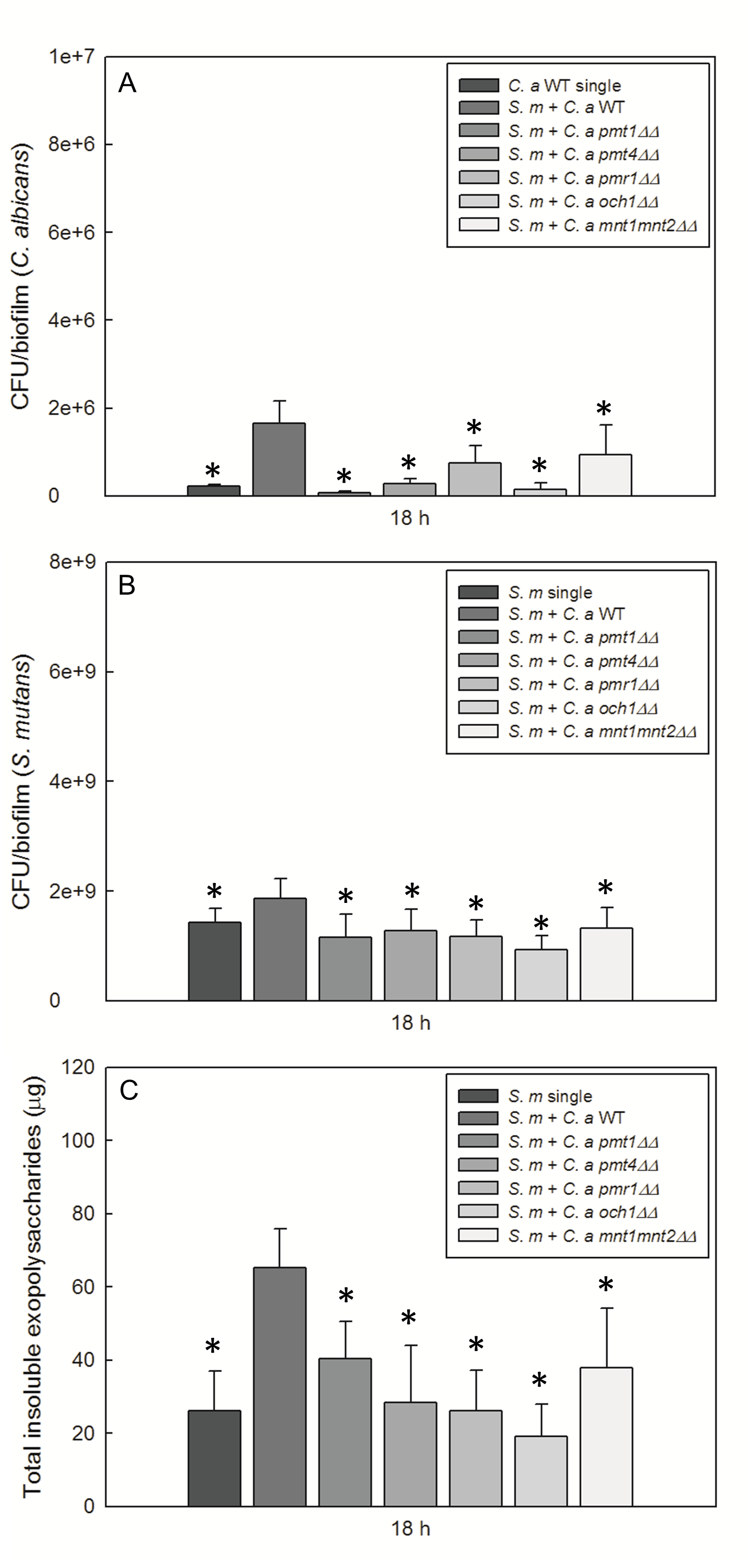

Supplement: S5 Fig — CFU of (A) C. albicans, (B) S. mutans, and (C) total insoluble EPS in mixed-species biofilms. Asterisk indicates that the values are significantly different from the mixed-species biofilm formed with S. mutans and C. albicans WT (P < 0.05). (TIF) [file ppat.1006407.s005.tif]

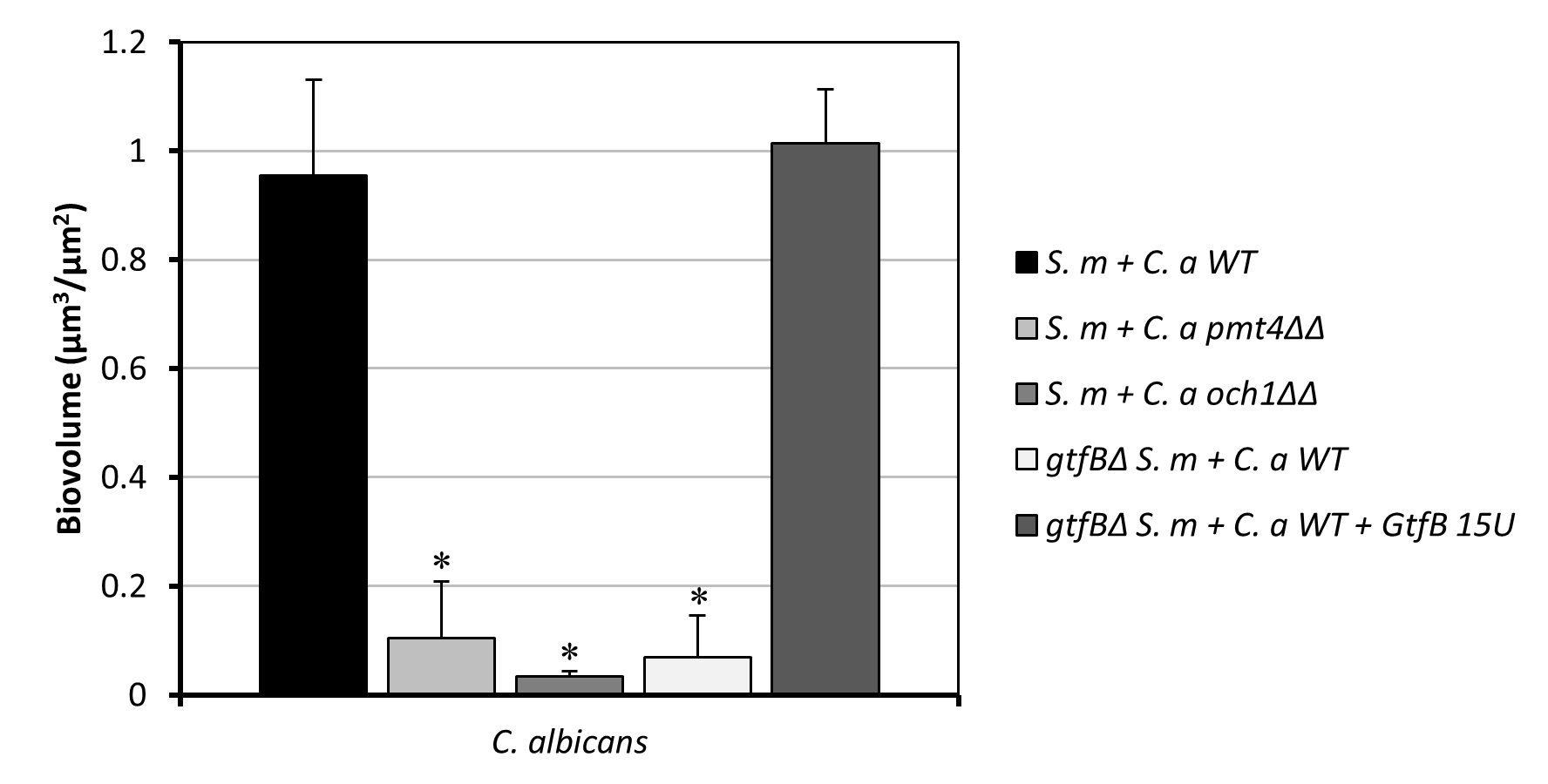

Supplement: S6 Fig — Asterisk indicates that the values are significantly different from the mixed-species biofilm formed with S. mutans and C. albicans WT or the one with ΔgtfB S. mutans and C. albicans WT supplemented with 15 U of GtfB (P < 0.05). (TIF) [file ppat.1006407.s006.tif]

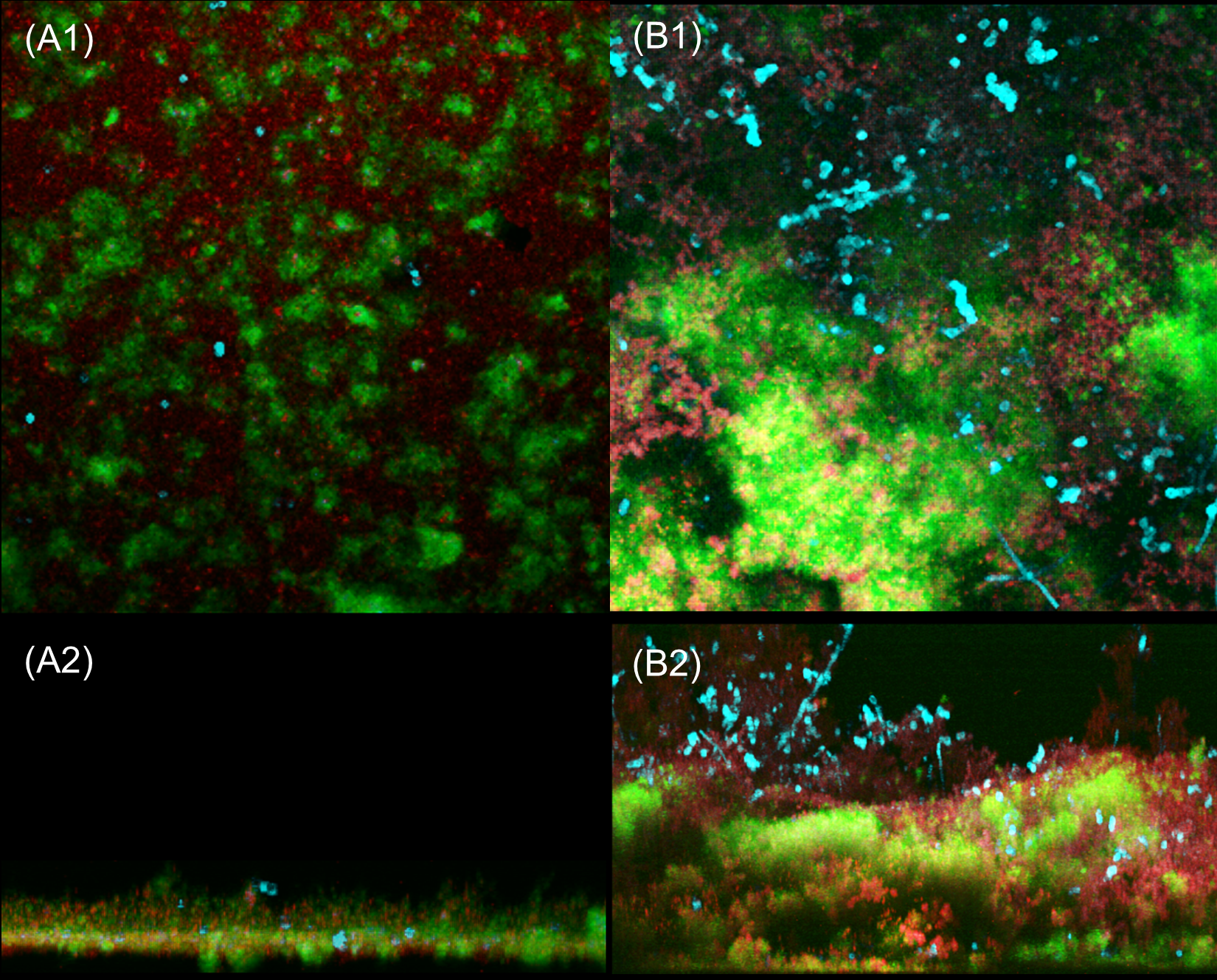

Supplement: S7 Fig — (A1) Top view and (A2) orthogonal views of biofilms with no GtfB supplemented; (B1) top view and (B2) orthogonal views of biofilms with 15 U of GtfB supplemented. (TIF) [file ppat.1006407.s007.tif]

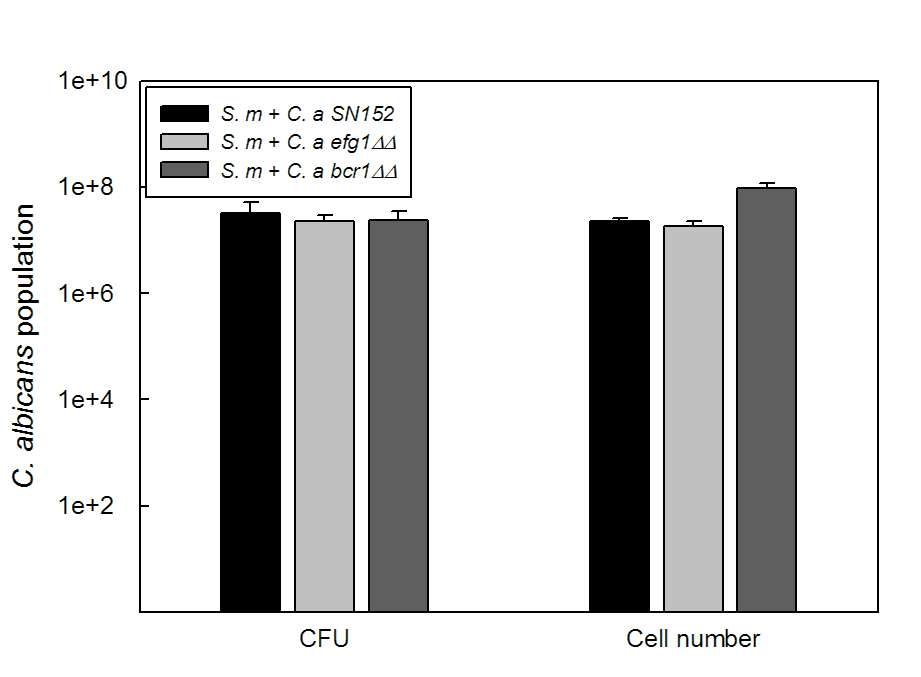

Supplement: S8 Fig — There are no significant differences in both CFU and cell number between C. albicans wild type and mutant strains. (TIF) [file ppat.1006407.s008.tif]

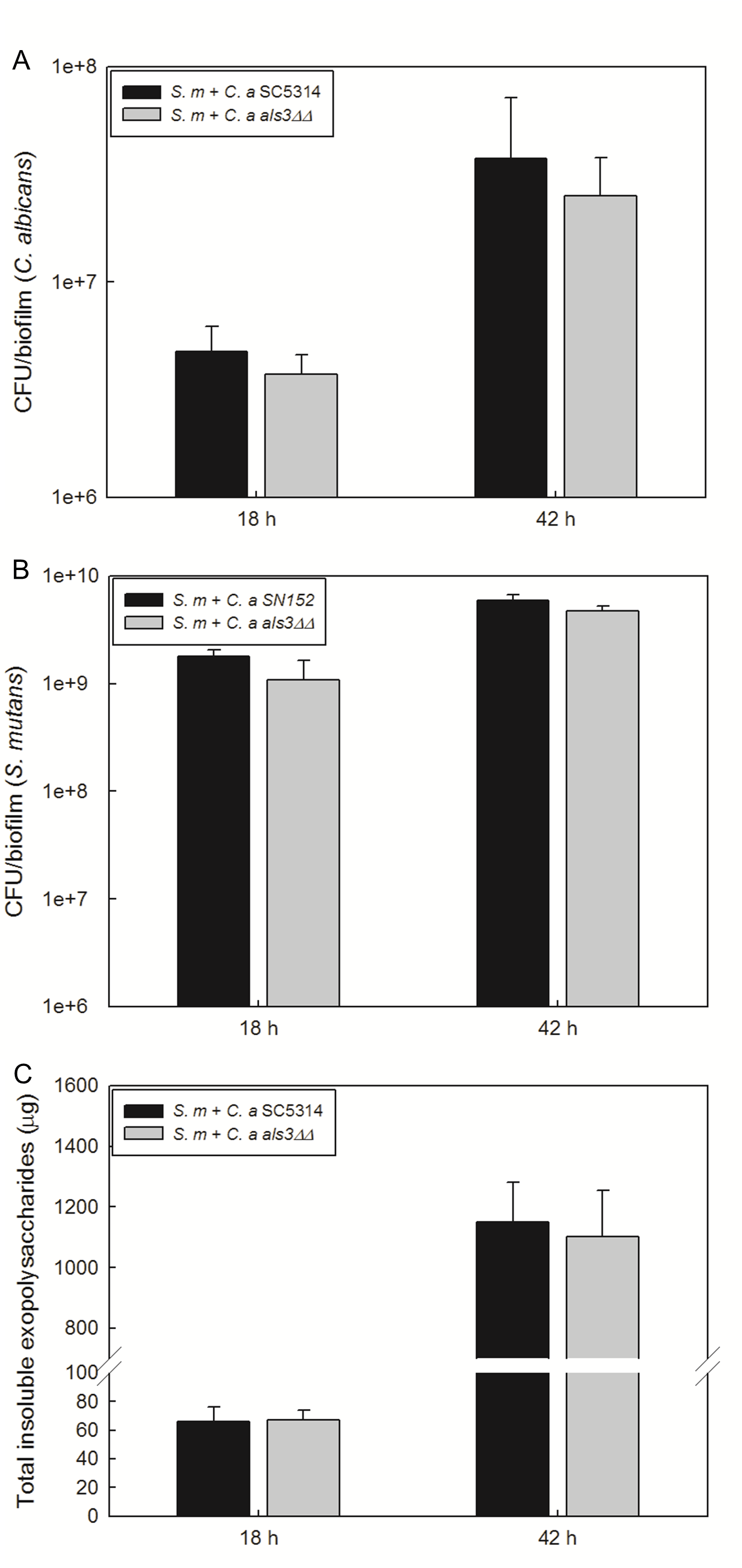

Supplement: S9 Fig — CFU of (A) C. albicans, (B) S. mutans, and (C) total insoluble EPS in mixed-species biofilms. In our model, C. albicans defective in ALS3 expression was capable of developing robust mixed-species biofilm with S. mutans. (TIF) [file ppat.1006407.s009.tif]

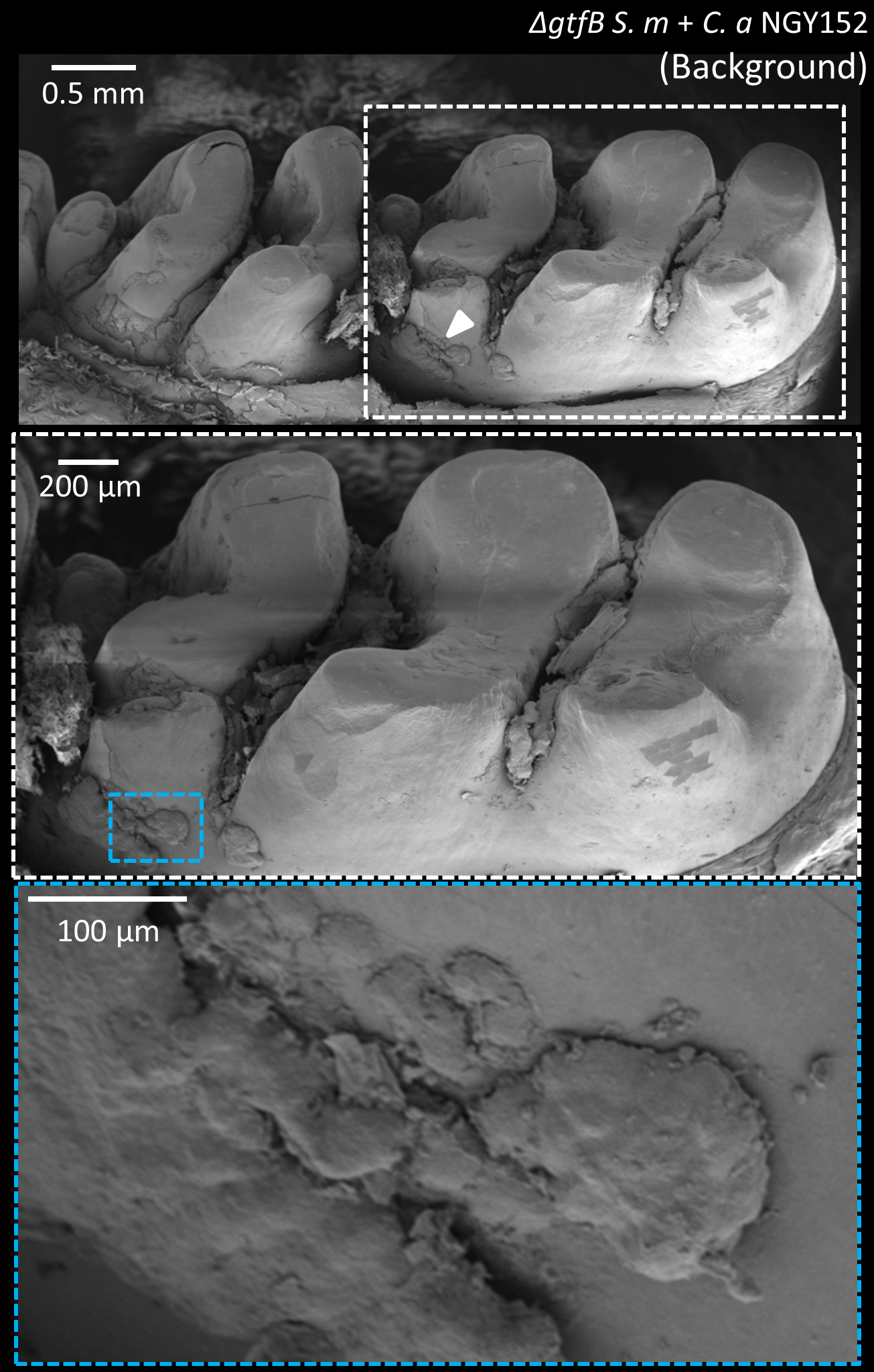

Supplement: S10 Fig — (TIF) [file ppat.1006407.s010.tif]

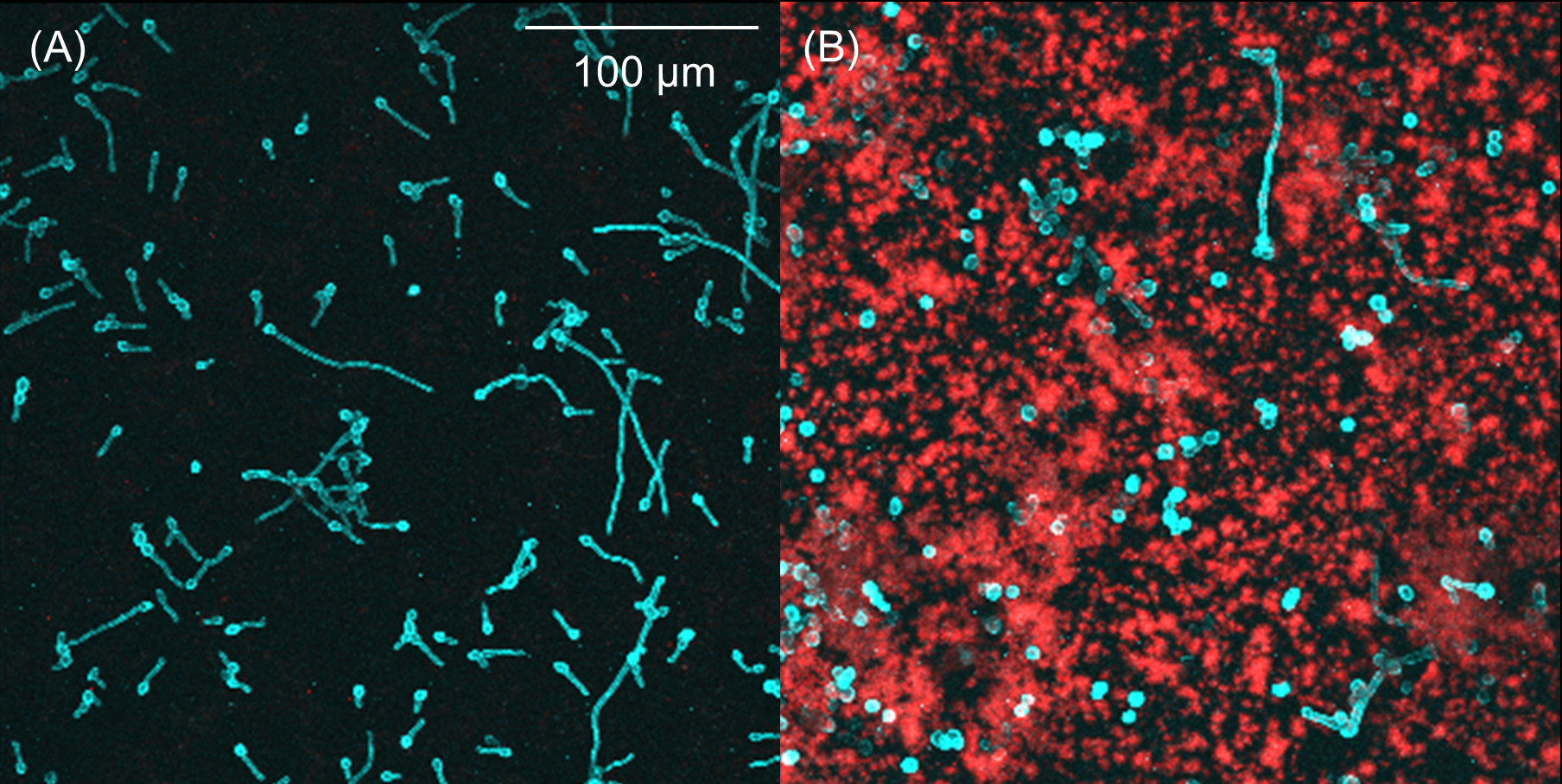

Supplement: S11 Fig — (A) C. albicans single-species biofilm showed no labeling of fungal β-glucan in the absence of GtfB, (B) while abundant labelled glucan (in red) was observed when GtfB (15 U) was added during C. albicans biofilm formation, demonstrating highly specific labeling of α-glucans produced by GtfB. (TIF) [file ppat.1006407.s011.tif]
